# A Study Based on Metabolomics, Network Pharmacology, and Experimental Verification to Explore the Mechanism of Qinbaiqingfei Concentrated Pills in the treatment of Mycoplasma Pneumonia

**DOI:** 10.3389/fphar.2021.761883

**Published:** 2021-11-04

**Authors:** Zheng Liu, Jin-hai Huo, Wen-ting Dong, Guo-dong Sun, Feng-jin Li, Ya-nan Zhang, Zhi-wei Qin, Jiang pengna, Wei-ming Wang

**Affiliations:** ^1^ Heilongjiang Academy of Chinese Medicine, Institute of Chinese Materia Medica, Harbin, China; ^2^ Heilongjiang University of Chinese Medicine, Harbin, China

**Keywords:** Qinbaiqingfei concentrated pills, metabolomics, network pharmacology, NF-κB signaling pathway, mycoplasma pneumonia

## Abstract

Qinbaiqingfei concentrated pills (QB) are a commonly used medicine for the treatment of mycoplasma pneumonia in China, and the mechanism of action of QB needs to be studied further. Therefore, we use a combination of metabolomics and network pharmacology to clarify the mechanism of QB. Nontarget metabolomics studies were performed on rat serum, urine, and lung tissues, and 56 therapeutic biomarkers were found. Subsequently, the components of QB absorbed into the blood and lung tissues were clarified, and based on this finding, the core target of network pharmacology was predicted. The enrichment analysis of biomarkers–genes finally confirmed their close relationship with the NF-κB signaling pathway. By western blotting expression of the proteins in the lung tissue–related signaling pathways, it is finally confirmed that QB inhibits the NF-κB signaling pathway through SIRT1, IL-10 and MMP9, CTNNB1, EGFR, and other targets. It plays a role in regulating immunity, regulating metabolism, and treating diseases.

## Introduction

*Mycoplasma pneumoniae* (MP) infection is a common source of community-acquired infection. *Mycoplasma pneumoniae* pneumonia (MPP) is a common infectious disease in children (Alishlash et al., 2019). Current evidence shows that the incidence of MPP in children over 4 years of age is significantly higher than that in other age groups (Waites et al., 2017). Researchers have found that MPP infection is closely related to climatic changes (Onozuka et al., 2009; Xing et al., 2020). Its outbreak can cause serious community infections (Bajantri et al., 2018). Although MPP is a self-limited disease with pulmonary symptoms occurring in only about 3–13% of patients (Saraya, 2017), about 3.5 million people have died of mycoplasma pneumonia (World Health Organization) every year since it was first reported clinically by Dr. Reimann in 1938.

MP, as the smallest pathogen currently discovered, can activate the immune process by inducing tissue damage through adhesion to host epithelial cells (Atkinson et al., 2008). Adhesion to cells surfaced by adhesin P30 and P1 protein prevents removal (Waites et al., 2008). Next, the release of superoxide anions and toxin CARDS inhibited the cell H_2_O_2_ enzyme to cause oxidative damage, cause host cell vacuolation, and induce cell apoptosis (Almagor et al., 1984; Kannan and Baseman, 2006). This is followed by the activation of the immune system, including toll-like receptor excitation in the cell membrane (Naghib et al., 2018), which triggers a cascade of protein signals downstream. We have a reason to believe that these receptors recognize MP through pathogen-associated molecular patterns and endogenous damage–associated molecular patterns. On the one hand, the receptors trigger the body’s metabolic disorder. On the other hand, they induce the expression of pro-inflammatory factors, cause inflammatory infiltration, and recruit white blood cells to induce inflammation (Karimi and Arababadi, 2014; Naghib et al., 2018; Hsieh et al., 2020). By changing the levels of cytokines, MPP can significantly change Th1/Th2 levels (Yang et al., 2019). Current research supports these claims and explains the causes of extrapulmonary symptoms. MPP is always like a hidden ghost trying to avoid activating the innate immune system (Merrell and Falkow, 2004). This is related to its destructiveness and structure. It also reflects the fact that asymptomatic infections of MPP account for the majority of cases (Bajantri et al., 2018), but the proportion of hospitalized patients and the mortality rate is gradually increasing (Khoury et al., 2016). The symptoms of MPP patients are mostly respiratory diseases, accompanied by flu-like symptoms such as fever, headache, cough, and muscle aches. There are often pulmonary rales and lung base dullness (Kumar, 2018). In severe cases, it leads to diffuse pneumonia, respiratory distress syndrome, etc. (Kannan et al., 2012). Because of the influence of inflammatory factors of the dimer of complement and fibrin-D, it causes long-lasting immune damage and causes vasculitis, which leads to thrombosis. This change will affect the entire body and cause harm including encephalitis (Lin et al., 2014). MP secretion stimulates the ganglia to cause permanent damage to the peripheral nervous system and central nervous system (Narita, 2009).

To treat MPP, clinically effective macrolides are commonly used (Blyth and Gerber, 2018). However, mild patients and children should try to avoid antibiotics. In East Asia, the drug resistance rate of macrolides is increasing year by year (Liu et al., 2009; Zhou et al., 2014; Yamazaki and Kenri, 2016; Yang et al., 2017). Research on the resistance of lactams is also ongoing (Purba et al., 2019; Liu et al., 2020).

Traditional Chinese medicine is guided by the theories of traditional Chinese medicine, which is widely used in East Asia and Southeast Asia and has outstanding efficacy (Li, 2020). Qinbaiqingfei concentrated pills are made by Baikal skullcap (Pharmacopoeia of China 2015), Pheretima (Pharmacopoeia of China 2020), Stemonae Radix (Pharmacopoeia of China 2015), Asteris Radix et Rhizoma (Pharmacopoeia of China 2015), Ophiopogon (US FDA Substance Registration System 2016) and Platycodonis Radix (Pharmacopoeia of China 2015). It is the first traditional medicine approved by the Chinese government for the clinical treatment of mycoplasma pneumonia (2004 L04185). We have confirmed that baicalin and platycodin D in QB is produced by inhibiting MP adhesin protein P1 and acting on the epidermal growth factor. Antimycoplasma ability and excellent tissue repair function (Meng et al., 2012; Meng et al., 2014; Meng et al., 2017). However, our focus is always on specific points of action. This kind of inquiry is not suitable for initial research on natural medicines. Using systemic pharmacology and preliminary research, medicine is also a focus of our scientific research.

**FIGURE 1:**
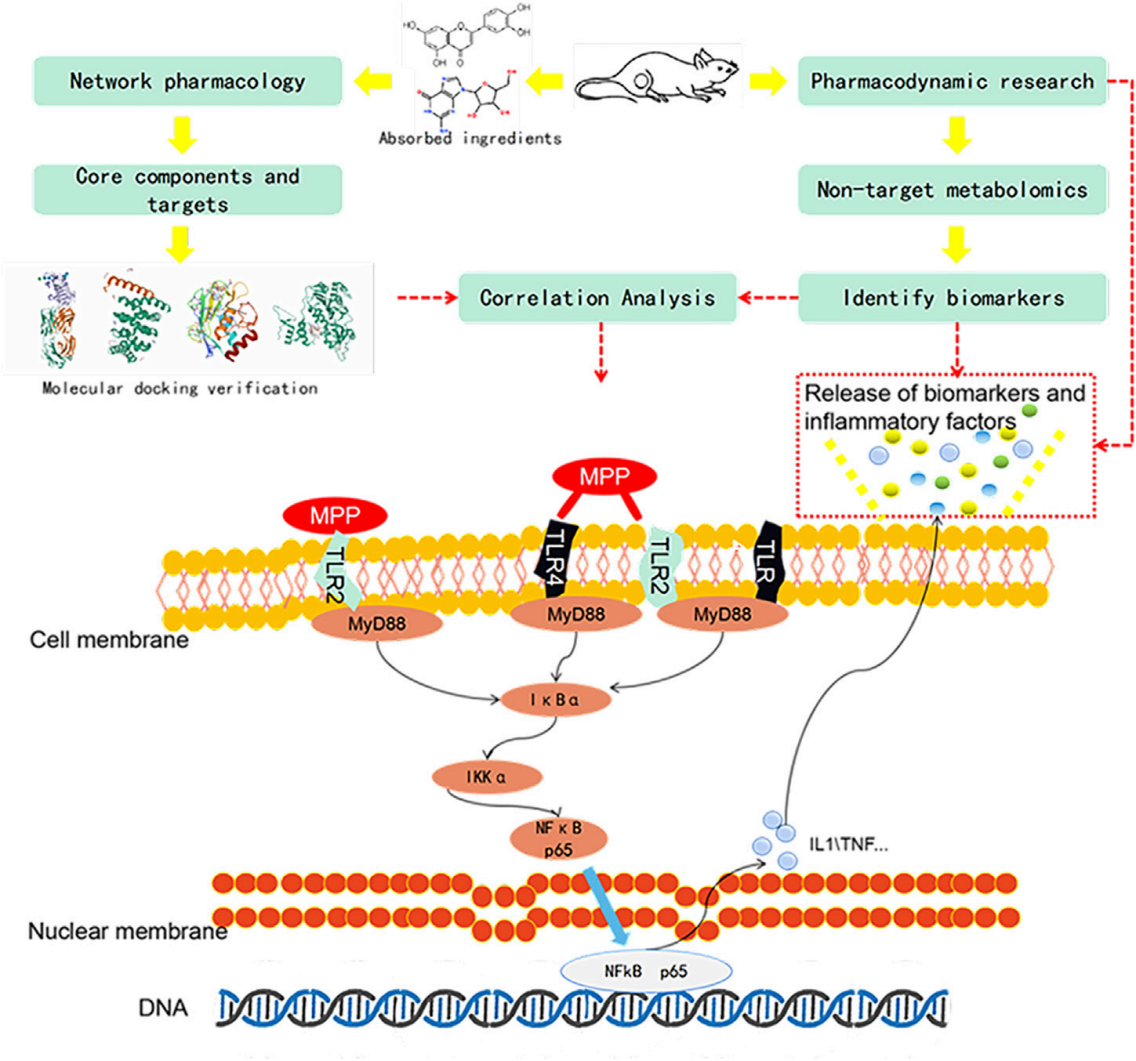
Process design diagram.

Although advances in technology have helped us discover countless new disease targets, we still face the agony of many diseases and are powerless to prevent them. Explaining the mechanism of action of complex drugs is always a difficult subject. Combined with biomarker screening, pharmacodynamics, and pharmacokinetics, research has become a direction of our current concern (Collis, 2006). Firstly, we use pathological slices and an inflammation level detector to evaluate the efficacy. Secondly, the UPLC-Q/TOF-MS technology is used to perform untargeted identification and analysis of the collected samples, explaining the potential interactions between various endogenous metabolic processes and exogenous effects of diseases or drug treatments. To analyze the changes of biomarkers, find out interpreting the biological significance of the potentially affected biomarkers (Qian et al., 2018). Network pharmacology can be used to predict the correlation between targets and indications. The molecular docking technology that simulates the molecular structure at the molecular level and the dynamic level verifies the binding status between the targets (Tang et al., 2020). Through the integrated analysis of them, we explore its mechanism of action in the treatment of mycoplasma pneumonia. Figure 1.

## Materials and Methods

### Ethical Approval

This study was reviewed by the Ethics Committee of the Heilongjiang Academy of Chinese Medicine Sciences (No. 2015-64) and the experiments were conducted by the Chinese Government’s “Guidelines on the Care and Use of Laboratory Animals” to minimize their suffering.

### Reagents and Materials

Reagents included Qinbaiqingfei concentrated pills (Heilongjiang Academy of Chinese Medicine, 20200715 China), azithromycin (Pfizer, United States), methanol, acetonitrile (chromatography pure, Merck, Germany), formic acid (chromatography pure, Fisher, United States), distilled water (Watsons, China), methanol, acetonitrile (analysis of pure, Kemiou, China), the rat serum IL-10, TNF-α ELISA kit (Boster, China), mycoplasma pneumoniae nucleic acid detection kit (Da-an gene, China), NF-κB p65 rabbit polyclonal antibody, IKK-α rabbit polyclonal antibody, IκB-α rabbit polyclonal antibody, MyD88 rabbit polyclonal antibody, β-actin antibody, and horseradish peroxidase–labeled goat anti-rabbit IgG (H+L) (Beyotime Biotechnology, China).

Materials included Acquity UPLC (Waters, United States, including binary high-pressure gradient pump, vacuum degasser, autosampler, and column oven), AB SCIEX Triple-TOFTM 5600^+^ mass spectrometer (AB SCIEX, United States, equipped with ESI Source and APCI source), data acquisition software was analyst TF 1.6 software (AB SCIEX, United States), data processing software system: Peakview 2.0/masterview1.0 software (AB SCIEX, United States), Progenesis QI 2.0 software (Waters, United States), Waters Acquity UPLC BEH C_18_ column (1.7 μm, 2.1 mm × 100 mm), Aquity UPLC BEH C_18_ VanGuard Pre-Column (1.7 μm, 2.1 mm × 5 mm), Bioer Line gene 9600 fluorescence Quantitative PCR instrument (Hangzhou Bioeri, China), Infinite M200 PRO (Tecan, Switzerland), Orthopolar photographic microscope (Nikon, Japan), and Amersham Imagery 600 gel imaging system (GE Company, United States).

### Experimental Animals

60 SPF Waster rats were raised in a constant temperature and humid environment. After 7 days of adaptive rearing, the rats were randomly divided into groups. Rats were anesthetized with pentobarbital during model building. Using the blind insertion method, the left lung cannula was inserted through the mouth and trachea to inject 200 μl of mycoplasma bacterial solution. The control group (CN) was injected with the same amount of sterile culture medium. For the next 6 days, the model was maintained by a 200 μl nasal drip after ether anesthesia every day. After modeling, the QB low-dose group (L) (0.8 g/kg) was administered intragastrically for 14 days, the QB medium-dose group (M) (1.6 g/kg), for 14 days and the QB high-dose group (H) (3.2 g/kg), for 14 days, and the azithromycin group (AZM) (45 mg/kg on the first day, 22.5 mg/kg), for 10 days. AZM and M correspond to adult doses. The adult dose of QB is 18 g/d, and the rat dose is calculated by the body surface area method. CN and MPP were given the same dose of purified water daily. On the last day, the rat’s urine was collected in a metabolic cage and then anesthetized with pentobarbital. The blood was taken from the abdominal aorta, and the supernatant was centrifuged after standing. The lung tissue was washed with normal saline, and the lower lobe of the right lung was fixed in formalin. Inside, the rest of the tissue is frozen in an ultra-low temperature refrigerator.

10 SPF Waster rats were reared in a constant temperature and humid environment. After being adaptively reared for 7 days, the rats were randomly divided into five groups. QB was given by intragastric administration at 16.2 g/kg, and CN was given equal volume of purified water. 1 h later, the rats were anesthetized with pentobarbital. Blood was collected from the hepatic portal vein and centrifuged, and the supernatant was frozen and stored. The lung tissue was washed and frozen.

### PCR Amplification of Mycoplasma

Take 100 mg rat lung tissue, add 100 μl of water to homogenize for 2 min, centrifuge at 6500 rpm for 10 min, take the supernatant, centrifuge at 12,000 rpm for 5 min, add 50 μl DNA extracted from the pellet, treat with 100°C for 10 min, centrifuge at 12,000 rpm for 10 min, and add 2 μl of the supernatant to the reaction tube. The amplification conditions were 93°C–2 min; 93°C, 45 s – 55°C, 60 s, 10 cycles; and 93°C 30 s – 55°C 45 s, 30 cycles.

### Pathological Treatment

The fixed lung tissue was washed with distilled water and dehydrated with ethanol gradient. Xylene was transparently treated twice, soaked in wax, and embedded in a paraffin embedding machine. The embedded tissue was fixed on a microtome for sectioning and placed on a clean carrier. On the glass slides, the slides are dried in a 60°C constant temperature oven, and then dewaxed, HE stained, dehydrated, and finally sealed with neutral gum.

### Biochemical Index Detection

After the rat serum was thawed, it was centrifuged at 2000 rpm for 10 min. The supernatant was taken for ELISA detection, and a microplate reader was used for detection.

### UPLC-Q/TOF-MS Sample Processing

Freeze urine samples are gradually heated and thawed; take 200 μl of urine, add 200 μl of distilled water, vortex and centrifuge at 4°C, 13,000 r/min for 10 min, and take the supernatant. Take 200 μl of the serum, add 800 μl of methanol (precooled at −20°C), vortex for 2 min, let it stand for 10 min, and centrifuge at 4°C, 13,000 r/min, 10 min. Take 800 μl of the supernatant, blow dry with nitrogen, add 200 μl of 80% methanol (precooled at −20°C) to reconstitute, vortex for 2 min, 4°C, 13,000r/min, centrifuge for 10 min, and take the supernatant to obtain. Take 50 mg of the lung tissue, add 0.25 ml of water for 2 min, add 1.5 ml of methanol: acetonitrile (1:1), vortex for 2 min, ultrasound for 2 min, and allow it to stand for 10 min. Then, take 1 ml of the supernatant and centrifuge at 13,000r/min for 10 min at 4°C, take 800 μl of the supernatant and dry it with nitrogen. Then, reconstitute it with 200 μl of 80% methanol, centrifuge at high speed, and collect the supernatant. Take a small amount of urine, serum, and lung tissue samples from each to make QC samples.

### Serum Pharmaceutical UPLC-Q/TOF-MS Sample Preparation

Take 400 μl of the rat blank serum and administration serum, add 10 μl of phosphoric acid to each, vortex for 60 s, add 2.4 ml methanol, vortex for 60 s at 4°C, 13,000 rpm for 10 min, blow-dry the supernatant with nitrogen and reconstitute the residue with 200 μl of methanol, vortex and shake for 60 s, and centrifuge at 13,000 rpm, 4°C for 10 min. Then, take the supernatant for UPLC-Q/TOF-MS analysis. Take 100 mg of the lung tissue, add 1 ml water, homogenize for 3 min, add 10 μl of phosphoric acid and vortex for 60 s, add 4 ml acetonitrile, vortex for 2 min, allow it to stand for 10 min, and centrifuge at 4°C, 13,000 rpm for 10 min.

### Mass Spectrometry Conditions

Adopt an ESI ion source, the ionization mode is electric spray positive and negative ion mode, the positive and negative ion source voltage is 5500 V/−4500 V, ion source temperature is 550°C, cleavage voltage is 100 V/−100 V, collision energy are 35 eV/−35 eV, and collision energy extension are 15 eV/−15 eV. The atomizing gas is nitrogen, the auxiliary gas Gas1 is 55 PSI, the auxiliary gas Gas2 is 55 PSI, and the curtain gas CurGas is 35 PSI. The scanning range of the precursor ion of the primary mass spectrum is 80–1200 Da. IDA sets the eight highest peaks with a response value of over 100 cps to scan the secondary mass spectrum; the scanning range of the product ion is 50–1200 Da, and dynamic background subtraction is enabled.

### The Chromatographic Conditions

Column temperature: 35°C; mobile phase: A: 0.1% formic acid water and B: 0.1% formic acid acetonitrile. The gradient elution sequence is as follows and only write the proportion of B. Urine: 5–45% from 0 to 16 min, 45–100% from 16 to 18 min, and 5% from 18.1 to 20 min. Serum: 5–55% from 0 to 5 min, 55–65% from 5 to 9 min, 65–75% from 9 to 13 min, 75–100% from 13 to 18 min, and 5% from 18.1 to 20 min. Lung tissue: 5–55% from 0 to 3 min, 55–65% from 3 to 6 min, 65% from 6 to 8 min, 65–70% from 8 to 10 min, 70–100% from 10 to 18 min, and 5% from 18.1 to 20 min. Blood components: 5–55% from 0 to 5 min, 55–65% from 5 to 9 min, 65–75% from 9 to 13 min, 75–100% from 13 to 18 min s, and 5% from 18.1 to 20 min. Lung component: 5–55% from 0 to 3 min, 55–65% from 3 to 6 min, 65% from 6 to 8 min, 65–70% from 8 to 10 min, 70–100% from 13 to 18 min, and 5% from 18.1 to 20 min. The flow rate is 0.3 ml/min and the injection volume is 5 μl.

### Liquid and Mass Data Processing

Import the collected metabolic profile data onto Progenesis QI software, and go through peak matching, peak extraction, normalization, data dimension reduction, and mass spectrum matrix information acquisition. Establish the variable projection importance VIP file. Select VIP>1 variables as potential biomarkers preliminary screening for these potential markers, further analysis of the trend of changes in the content of these potential markers for the sample. The second potential marker screening is performed based on the principle of significant differences in metabolites between groups (*p* < 0.05) to obtain potential biomarkers things. In this experiment, we first screened between the control group and the MPP group and preliminarily screened based on the principles of VIP>1 and *p* < 0.05. Then, we reviewed the initially selected compounds with the administration group to initially determine the difference.

Use the Masterview function in Peakview2.0 software to import the liquid quality acquisition data of the administered serum and the blank serum. Set the mass error <5 ppm; weight 30%, isotope difference <10%; weight 40%, Formula finder score >70%; weight 40%, sample/control is three times to deduct the interference of the blank serum endogenous components. Compare the remaining detected ions with the retention time, isotope kurtosis ratio, the accurate mass of the precursor ion, and the secondary mass spectrum of the compound detected in the extract of QB. If they are consistent, it is confirmed as the prototype component in QB.

### Component Targets and Disease Screening

Target screening of the obtained ingredients, in SwissTargetPrediction (principle: ligand structure similarity), DrugBank (principle: ligand structure similarity), PharmMapper (principle: pharmacophore matching), and BATMAN-TCM (principle: query component Possible component target interactions and the similarity of component target interactions known in the database). Set Probability ≥0.70 in SwissTargetPrediction, select “Homo sapiens” in “Choose an organism.” Select “0.70” in “Similarity threshold” in DrugBank, set “20” in BATMAN-TCM “Score cutoff,” and NormFit ≥0.70 in PharmMapper to get the final possible target.

In the eight databases of NCBI Gene, DrugBank, GeneCards, DisGeNET, TTD, PharmGKB, OMIM, and KEGG, search with “Pneumonia” and “Mycoplasma pneumonia” as keywords. In addition, the relevant gene chips were retrieved in GEO with “Pneumonia” and “Mycoplasma pneumonia” as keywords, and impute package v1.62.0 and limma package v3.44.3 of R v4.0.0 were used to impute missing values. Standardize processing and pass experience Bayesian model is used to determine the differential gene, and the differential gene threshold is set to *p* < 0.05 and |log2 fold change|≥1. In order to ensure the accuracy of disease target prediction, the disease targets of the abovementioned sources are intersected to obtain the disease target and the intersection target is obtained.

### C-T Network and T-D Network Construction and Topology Analysis

The contribution of the components to the C-T network is evaluated by introducing the Contribution Index. The PubMed search strategy is to use “Pneumonia” and “Mycoplasma pneumonia” in the Abstract/Title section. The English name of the active ingredient on the Internet and the commonly used keywords are jointly searched for documents from 1990 to December 1, 2020. Build a C-T network through screening.

The T-D network obtains the interaction between them through STRING. Set “organism” and “minimum required interaction score” to “Homo sapiens” and “medium confidence (>0.400).” Construct a T-D network by importing Cytoscape. Combine coreness, closeness, degree, average shortest path length, and betweenness to judge the importance of nodes. The filter condition is first> the mean degree. Then, it meets the mean values of degree, closeness, betweenness, and coreness at the same time and less than the mean value of the average shortest path length.

### Molecular Docking

For molecular docking of the core target and core components, use GEMDOCK and select “200,” “Population size,” “Generation,” “Number of solutions,” and “Default setting” in the software operation interface. “Docking Accuracy Settings.” “7,” “2,” and “Docking standard,” the other docking parameters, are all default.

### Biomarker and Core Target Enrichment Analysis

Using Progenesis QI 2.0 software, import the experimentally obtained disease treatment targets and biomarkers with significant callback effects to http://impala.molgen.mpg.de/ for comprehensive molecular pathway level analysis. Analyze genes and metabolites that carry out integration and association. Through the screening of the *p* value and Q value, the path with biological significance is screened to the limit of satisfying *p* < 0.05 and Q < 0.05 at the same time.

### Protein Expression Detection of Core Pathways

Take 50 mg of the rat lung tissue, add 500 μl of PBS and homogenize on ice bath, and centrifuge at 500 g for 3 min. Remove the supernatant, add 100 μl of the nucleoprotein extraction reagent and extract and centrifuge, take the supernatant, and add the loading buffer. It can be pyrolyzed and stored in aliquots. Prepare a separation gel and concentrated gel (accelerating coagulation at 37°C) in accordance with the requirements of the kit and load a small amount of the sample. Then use 80 mV for concentrated gel separation, 120 mV for separation gel separation, and 200 mA, 1.5 h for CN membrane transfer and membrane washing. It is then blocked with 5% skim milk for 0.5 h, blocked with the primary antibody overnight, and incubated with the secondary antibody for 2 h. After washing the membrane, add luminescent solution to take pictures.

## Results

### The Amount of Mycoplasma Detected by RT-PCR

**FIGURE 2:**
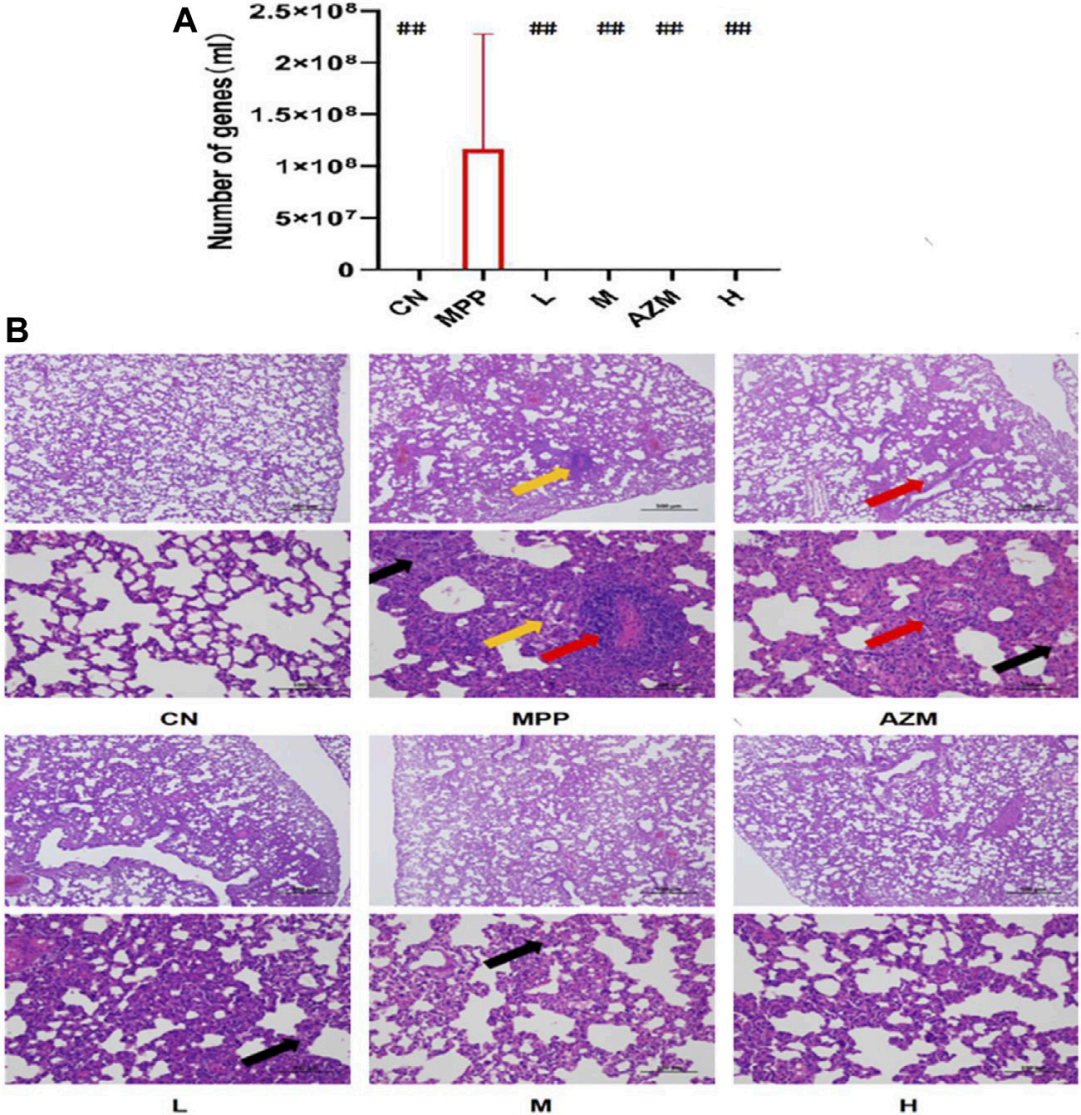
HE staining of rat lung tissue and the amount of mycoplasma carrier in lung tissue. **(A)** RtPCR mycoplasma real-time fluorescence detection, using analysis of variance to calculate the significant difference, compared with the MPP group #*p* < 0.05, ##*p* < 0.01. **(B)** The alveolar wall of the control group was composed of a single layer of epithelium with a simple structure; the interstitium, including the connective tissue and blood vessels in the lung, had no obvious abnormalities. In the *Mycoplasma pneumoniae* group, large areas of alveolar walls are severely thickened, alveolar sizes vary, alveolar walls are infiltrated with a large number of inflammatory cells (black arrows), and few of the foam cells in the alveolar cavity (yellow arrows) and local perivascular inflammatory cells infiltrate into rings (red arrow). In the QB group, the alveolar wall was slightly thickened, with a small amount of inflammatory cell infiltration in the alveolar wall (black arrow). In the AZM group, large areas of alveolar walls were moderately thickened, alveolar sizes varied, and alveolar walls were infiltrated with moderate inflammatory cells (black arrows).

The amount of mycoplasma in rats in the MPP group reached 1.15 × e^7^. Both the QB and AZM groups decreased, which was below the minimum detection limit of the kit (Figure 2A). In this case, it can be considered that there is no infectious ability.

### Histopathological Analysis

**FIGURE 3:**
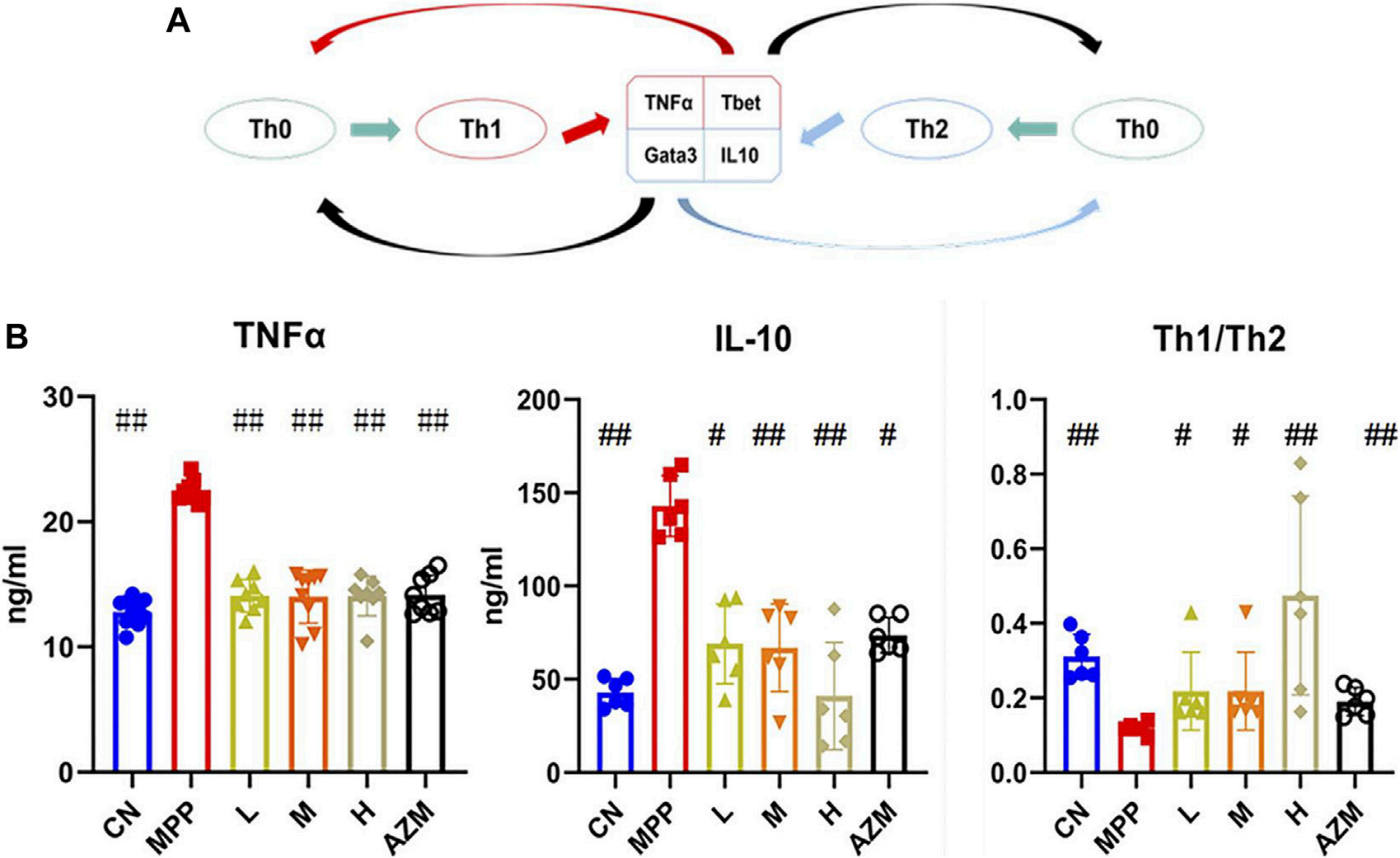
**(A)** T-cell differentiation diagram, the black arrow indicates inhibition and the remaining arrows indicate promotion. **(B)** Serum TNF-α and IL-10 expression levels, and the Th1/Th2 ratio. The significant difference was calculated using analysis of variance, #*p* < 0.05, ##*p* < 0.01 and compared with the MPP group.

A representative photomicrograph of HE staining is shown in Figure 3B. In the control group, the surface of the lung tissue was smooth and the alveolar wall structure was clear. In the pneumonia group, alveolar walls were thickened and eosinophils were occasionally increased. There are some foam cells and the inflammatory infiltration near the blood vessels forms a ring. These two conditions indicate that this has caused damage to the vascular structure. Both QB and AZM alveolar wall thickness decreased, and alveolar stenosis was not seen. The QB group recovered better, with tissue fluid exudation and inflammatory infiltration significantly contracted. This is related to its composition. We have confirmed that QB has good tissue repair ability (Meng et al., 2014), which is a unique advantage over AZM.

### Biochemical Index Determination

Using IL-10, TNF-α, and Th1/Th2 ratios as evaluation indicators (Figure 3A), the MPP, CN, QB, and AZM groups were collected to evaluate the effect of QB on MPP. Compared with the control group, the levels of IL-10 and TNF-α in the MPP group were significantly increased. The Th1/Th2 ratio showed an upward trend (*p* < 0.01). After QB and AZM treatment, the above indicators recovered (Figure 3B). It should be noted that the Th1/Th2 ratio is higher in the control group than in the pneumonia group, but IL-10 also has a rising trend in the MPP group. The differentiation of Th0 cells depends on the expression of the transcription factors T-bet and GATA-3 (Tibbitt et al., 2019). In the early stage of the disease, there is a Th1 advantage (Wu et al., 2019). With the development of the disease, the activation of innate immunity requires the activation of Th2 cells and is used to deactivate the precursor of IgE (Wynn, 2015). At the same time, it promotes the secretion of eosinophils, airway hyperresponsiveness, and mucus production (Hamilos, 2015). As a sensitizing inflammatory factor, long-term high expression of Th2 can not only inhibit the inflammatory response but also cause allergic tissue damage (Tibbitt et al., 2019). Th2 dominance exists in the later stage of the disease, as a means for the body to resist foreign invasion (Koh et al., 2001).

### Metabolic Profile Analysis

**FIGURE 4:**
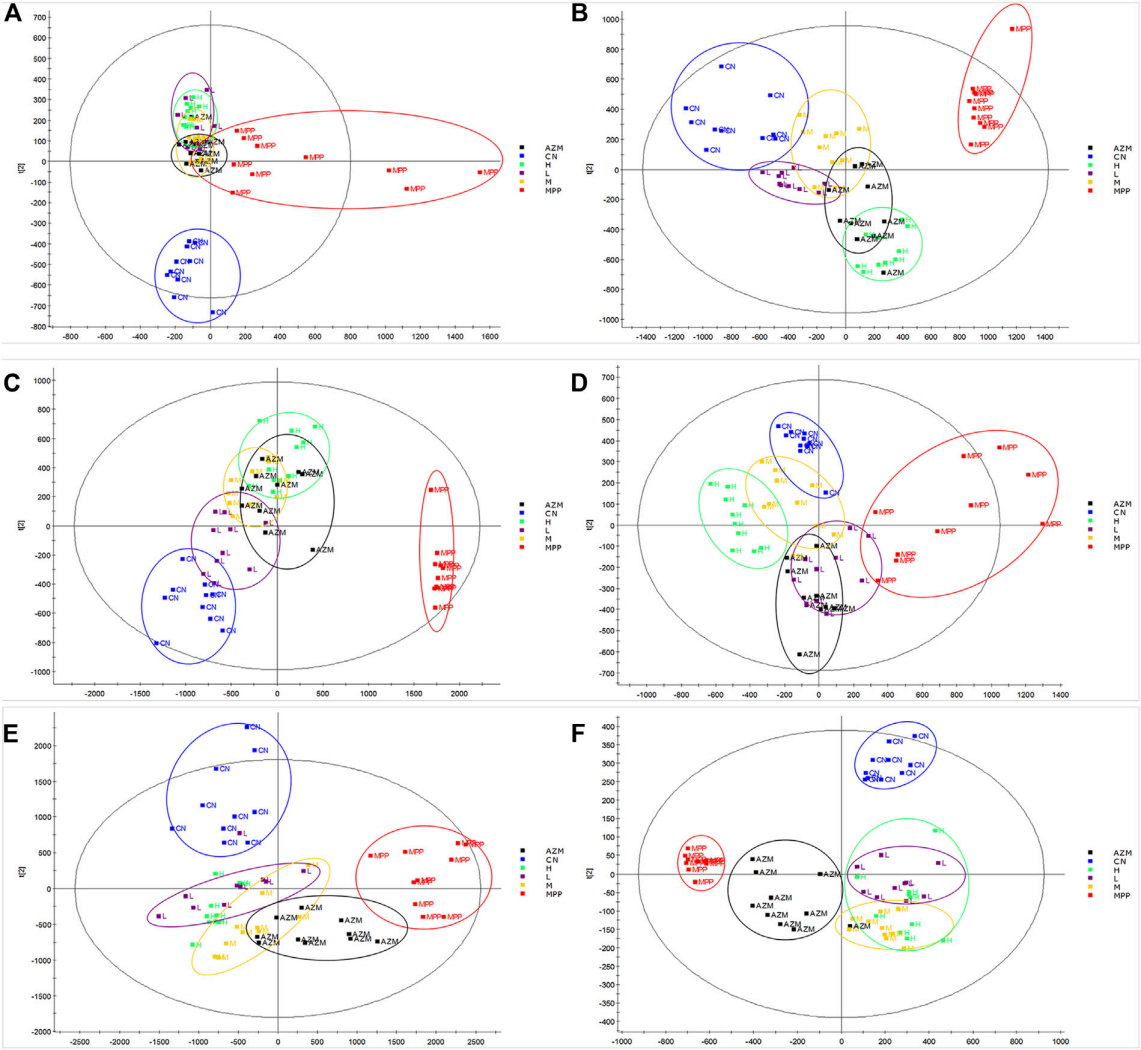
Urine, serum, and lung tissue OPLS-DA images of rats in CN, MPP, QB, and AZM groups. **(A)** Four groups of urine positive ion mode OPLS-DA diagram **(B)** Four groups of urine negative ion mode OPLS-DA diagram **(C)** Four groups of serum positive ion mode OPLS-DA diagram **(D)** Four groups of serum negative ion mode OPLS-DA diagram **(E)** Four groups of lung tissues with positive ion mode OPLS-DA **(F)** four groups of lung tissues with negative ion mode OPLS-DA. We select 10 samples for each group.

**FIGURE 5:**
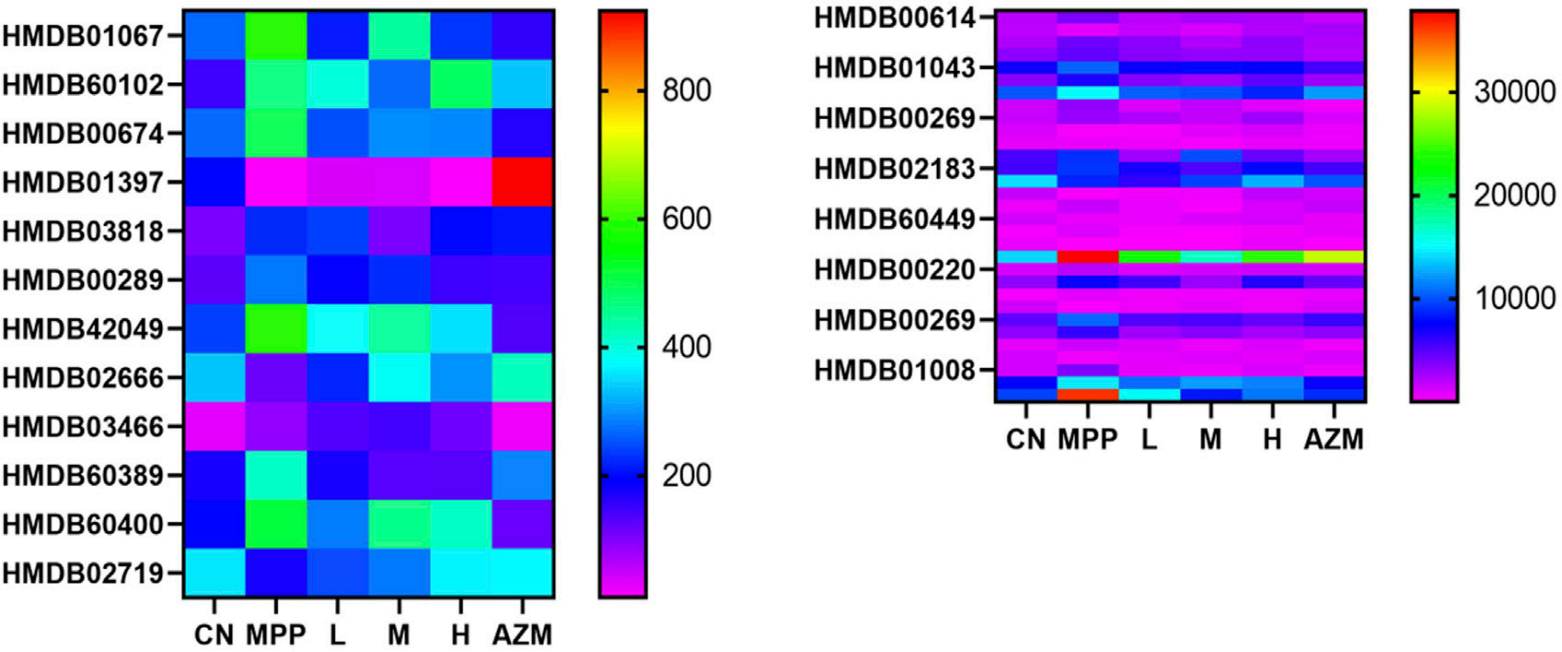
Urine, serum, and lung tissue have a tendency to recall the biomarker.

By constructing the OPLS-DA diagram, it is found that QB and MPP are well separated. QB and AZM are well separated from the MPP group and tend to be close to the CN group (Figure 4). MetaboAnalyst screened 105 compounds, and 56 were dosing callbacks (Table 1 and Figure 5).

**TABLE 1:** Urine, serum, and lung tissue have a tendency to pull back biomarkers.

No	Retention time(s)	M/S	M±H	HMDB	Chemical formula	Common name	Sources and trends	Max fold change
1	0.32	194.0476	M−H	HMDB01229	C_9_H_9_NO_4_	Dopaquinone	Serum↑##	6.6435
2	0.41	146.0255	M−H	HMDB06779	C_8_H_5_NO_2_	Indole-5,6-quinone	Serum↑##	7.7430
3	0.62	196.0633	M−H	HMDB00181	C_9_H_11_NO_4_	L-dopa	Serum↑##	4.7872
4	0.72	190.0523	M−H, M+FA−H	HMDB60400	C_10_H_9_NO_3_	5-phenyl-1,3-oxazinane-2,4-dione	Serum↑##	6.9405
5	0.74	182.0480	M−H	HMDB00017	C_8_H_9_NO_4_	4-pyridoxic acid	Serum↑##	5.1829
6	0.92	140.0668	M+Na	HMDB00043	C_5_H_11_NO_2_	Betaine	Serum↑#	2.3167
7	0.93	177.0403	M−H	HMDB03466	C_6_H_10_O_6_	L-Gulonolactone	Serum↑##	62.2394
8	0.96	167.0209	M−H	HMDB00289	C_5_H_4_N_4_O_3_	Uric acid	Lung↑##	2.5697
9	0.97	245.0308	M+FA−H	HMDB01268	C_8_H_8_O_6_	4-fumarylacetoacetic acid	Urine↓##	1.7411
10	1.61	150.0571	M+H	HMDB00696	C_5_H_11_NO_2_S	L-Methionine	Serum↓##	2.6644
11	2.01	283.0685	M−H	HMDB00299	C_10_H_12_N_4_O_6_	Xanthosine	Lung↓##	3.1764
12	2.09	686.1447	M−H	HMDB01373	C_21_H_35_N_7_O_13_P_2_S	Dephospho-CoA	Lung↓##	10.2279
13	2.76	228.0876	M+FA−H	HMDB00819	C_9_H_13_NO_3_	Normetanephrine	Serum↑##	2.3080
14	2.85	322.9500	M−H, 2M−H	HMDB42049	C_8_H_11_Cl_3_O_7_	Trichloroethanol glucuronide	Lung↑##	1.7335
15	3.47	291.0080	M−H	HMDB60449	C_7_H_15_Cl_2_N_2_O_4_P	Carboxyphosphamide	Urine↓##	1.4315
16	3.52	295.1261	M−H	HMDB60472	C_18_H_17_FN_2_O	Didemethylcitalopram	Urine↓#	1.5271
17	3.73	192.0665	M−H	HMDB60389	C_10_H_11_NO_3_	4-hydroxy-5-phenyltetrahydro-1,3-oxazin-2-one	Serum↑#	5.2162
18	3.84	343.0622	M−H	HMDB02666	C_12_H_17_N_4_O_4_PS	Thiamine monophosphate	Urine↓##	3.0919
19	4.09	693.4744	M+FA−H	HMDB00674	C_35_H_69_O_8_P	PA(16:0/16:0)	Lung↑##	1.8995
20	5.37	583.2534	M+H, M+Na	HMDB01008	C_33_H_34_N_4_O_6_	Biliverdin	Lung↑##	5.2458
21	5.45	391.2129	M+FA−H	HMDB04030	C_21_H_30_O_4_	21-Deoxycortisol	Serum↑##	1.8852
22	5.53	488.2968	M+H, M+Na, 2M+H	HMDB00138	C_26_H_43_NO_6_	Glycocholic acid	Serum↑##	3.0544
23	6.09	797.6591	2M−H	HMDB00222	C_23_H_45_NO_4_	L-Palmitoylcarnitine	Serum↑##	3.4013
24	6.28	318.2993	M+H	HMDB04610	C_18_H_39_NO_3_	Phytosphingosine	Serum↑##	3.4975
25	6.48	583.2534	M+H, M+Na	HMDB01008	C_33_H_34_N_4_O_6_	Biliverdin	Serum↑##	6.4105
26	6.50	377.1452	M+H, M+Na	HMDB00244	C_17_H_20_N_4_O_6_	Riboflavin	Urine↑#	1.7993
27	6.71	302.3047	M+H	HMDB00269	C_18_H_39_NO_2_	Sphinganine	Lung↑##	2.0649
28	6.74	335.2228	M+FA-H	HMDB03818	C_19_H_30_O_2_	5-androstenediol	Lung↑#	2.7749
29	6.83	395.2439	M+FA−H	HMDB00268	C_21_H_34_O_4_	Tetrahydrocorticosterone	Serum↑#	20.7376
30	6.96	539.0154	2M−H	HMDB06343	C_7_H_14_N_2_O_4_Se	Selenocystathionine	Urine↓##	4.0243
31	7.30	279.2306	M+Na	HMDB00220	C_16_H_32_O_2_	Palmitic acid	Serum↑##	1.8685
32	7.31	279.2312	M+H	HMDB01388	C_18_H_30_O_2_	Alpha-linolenic acid	Lung↓#	3.9947
33	7.40	302.3044	M+H	HMDB00269	C_18_H_39_NO_2_	Sphinganine	Serum↑##	4.5389
34	7.45	159.0447	M−H	HMDB60497	C_10_H_8_O_2_	Naphthalene-1,2-diol	Urine↓##	4.6030
35	7.56	319.2280	M−H	HMDB60102	C_20_H_32_O_3_	Arachidonate	Lung↑##	3.7587
36	7.74	303.0830	M−H	HMDB01067	C_11_H_16_N_2_O_8_	N-Acetylaspartylglutamic acid	Urine↓##	2.2912
37	7.97	522.3542	M+H	HMDB02815	C_26_H_52_NO_7_P	LysoPC(18:1(9Z))	Lung↑##	1.8640
38	8.11	303.2314	M+H, 2M+H	HMDB01999	C_20_H_30_O_2_	Eicosapentaenoic acid	Lung↑##	2.2904
39	8.35	417.1798	M+FA−H	HMDB03134	C_16_H_28_N_4_O_4_S	Biocytin	Urine↑##	1.5952
40	8.96	251.1994	M+Na	HMDB00806	C_14_H_28_O_2_	Myristic acid	Serum↑#	2.9917
41	10.34	301.2175	M−H	HMDB01999	C_20_H_30_O_2_	Eicosapentaenoic acid	Serum↑##	2.6415
42	11.46	357.0614	M+FA−H	HMDB06552	C_17_H_12_O_6_	Aflatoxin B1	Urine↓##	20.4369
43	11.64	279.2309	M+H	HMDB01388	C_18_H_30_O_2_	Alpha-linolenic acid	Serum↓##	7.9317
44	11.91	363.0159	M−H	HMDB60416	C_10_H_13_N_4_O_7_PS	6-thioinosine-5′-monophosphate	Urine↓##	1.6801
45	11.99	510.3905	M+H, M+Na	HMDB11149	C_26_H_56_NO_6_P	LysoPC(O-18:0)	Lung↑##	1.8216
46	13.43	327.2334	M−H	HMDB02183	C_22_H_32_O_2_	Docosahexaenoic acid	Lung↑##	3.7972
47	13.90	305.2472	M+H	HMDB01043	C_20_H_32_O_2_	Arachidonic acid	Lung↑#	1.4143
48	14.47	383.3293	M+H	HMDB03896	C_27_H_42_O	7-dehydrodesmosterol	Serum↓#	5.8721
49	15.06	327.2337	M−H	HMDB02183	C_22_H_32_O_2_	Docosahexaenoic acid	Serum↑##	2.0005
50	15.20	573.2078	M−H	HMDB06825	C_24_H_30_N_8_O_9_	Tetrahydrofolyl-[Glu](2)	Urine↓#	3.7036
51	15.28	385.3445	M+H	HMDB02719	C_27_H_44_O	Desmosterol	Serum↓##	5.7013
52	15.48	319.2282	M−H	HMDB60102	C_20_H_32_O_3_	Arachidonate	Serum↑##	1.9411
53	15.48	303.2339	M−H	HMDB01043	C_20_H_32_O_2_	Arachidonic acid	Serum↑##	3.1049
54	15.78	734.5678	M+H	HMDB00564	C_40_H_80_NO_8_P	PC(16:0/16:0)	Lung↑##	4.0799
55	17.36	281.2495	M−H	HMDB00207	C_18_H_34_O_2_	Oleic acid	Serum↑##	4.5778
56	17.85	302.3044	M+H	HMDB00269	C_18_H_39_NO_2_	Sphinganine	Urine↑##	3.2284

Compared with the MPP group, ↑ means increase, ↓ means decrease, #*p* < 0.05, ##*p* < 0.01.

### UPLC-Q/TOF-MS Data of Lung Tissue and Serum

Data collection of the rat serum and lung tissue was conducted, and 31 prototype components belonging to Qinbaiqingfei concentrated pills were identified. The results are shown in Table 2.

**TABLE 2:** Composition of QB into the blood and lung tissue.

No	Rt(s)	Common name	Chemical formula	M±H	Predicted value	Actual value	Error (ppm)	Fragment ion
1	1.75	Malic acid	C_4_H_6_O_5_	M+Na	157.01074	157.01211	8.7	157[M+Na]^+^,139[M+Na−H_2_O]^+^,111[M−H_2_O−CO]^+^
2	1.86	Casticin	C_19_H_18_O_8_	M+H	375.10744	375.10801	1.5	375[M+H]^+^,243[M+H−C_8_H_4_O_2_]^+^,110[M+H−CH_3_−C_12_H_10_O_6_]^+^
3	1.87	Astin B	C_25_H_33_N_5_O_7_Cl_2_	M+Na	608.16493	608.16795	5.0	608[M+Na]^+^,231[M+Na−C_14_H_16_N_3_O_5_Cl_2_]^+^
4	1.98	Eriodictyol	C_15_H_12_O_6_	M+H	289.07066	289.07032	−1.2	289[M+H]^+^,271[M+H−H_2_O]^+^,217[M+H−4H_2_O]^+^
5	2.52	Tuberospironine	C_18_H_27_NO_5_	M+H	338.19620	338.19617	−0.1	338[M+H]^+^,320[M+H−H_2_O]^+^,292[M+H−H_2_O−CO]^+^,264[M+H−C_3_H_6_O_2_]^+^,252[M+H−C_4_H_6_O_2_]^+^,236[M+H−C_4_H_6_O_3_]^+^
6	2.66	Stemotinine	C_18_H_25_NO_5_	M+H	336.18055	336.18075	0.6	336[M+H]^+^,308[M+H−CO]^+^,238[M+H−CO−C_4_H_7_O]^+^,222[M+H−C_5_H_7_O_2_−O]^+^,152[M+H−C_5_H_7_O_2_−C_4_H_6_O_2_]^+^
7	2.79	Tuberostemoline	C_22_H_31_NO_7_	M+H	422.21733	422.21698	−0.8	422[M+H]^+^,376[M+H−CH_2_O_2_]^+^,336[M+H−C_4_H_6_O_2_]^+^
8	2.87	Trans-Caffeic acid	C_9_H_8_O_4_	M+H	181.04954	181.04926	−1.5	181[M+H]^+^,152[M+H−CHO]^+^
9	3.13	Croomine	C_18_H_27_NO_4_	M+H	322.20129	322.20199	2.2	322[M+H]^+^,248[M+H−C_3_H_6_O_2_]^+^,162[M+H−2C_3_H_6_O_2_]^+^,136[M+H−2C_3_H_6_O_2_−C_2_H_4_]^+^
10	3.20	Chlorogenic acid	C_10_H_10_O_4_	M−H	193.05063	193.05080	0.9	193[M−H]^−^,149[M−H−H2O−CO]^−^,121[M−H−H_2_O−2CO]^−^
11	3.24	Asterin A	C_25_H_33_N_5_O_8_	M−H	530.22564	530.22387	−3.3	530[M−H]^−^,486[M−H−CH_2_NO]^−^,251[M−H−C_14_H_15_O_6_]^−^
12	3.45	Sessillistemonamine A	C_22_H_33_NO_5_	M+H	392.24315	392.24299	−0.4	392[M+H]^+^,318[M+H−C_4_H_10_O]^+^
13	3.51	Mirificin	C_26_H_28_O_13_	M-H	547.14571	547.14649	1.4	547[M−H]^−^,457[M−H−5H_2_O]^−^,367[M−H−C_6_H_12_O_6_]^−^,337[M−H−C_6_H_12_O_6_−CH_2_O]^−^
14	3.51	Astin J	C_25_H_33_O_7_N_5_	M-H	514.23072	514.22924	−2.9	514[M−H]^−^,484[M−H−2CH_3_]^−^,251[M−H−C_12_H_13_N_3_O_4_]^−^
15	3.68	Isorhamnetin	C_16_H_12_O_7_	M+H	317.06558	317.06503	−1.7	317[M+H]^+^,136[M+H−C_9_H_9_O_4_]^+^,111[M+H−C_10_H_6_O_5_]^+^
16	3.69	Wogonin	C_16_H_12_O_5_	M+H	285.07575	285.07575	0	285[M+H]^+^,270[M+H−CH_3_]^+^,253[M+H-CH_4_O]^+^,225[M+H−C_2_H_4_O_2_]^+^,213[M+H−C_3_H_4_O_2_]^+^,197[M+H−C_3_H_4_O_3_]^+^,137[M+H−C_3_H_4_O_2_−C_6_H_4_]^+^
17	3.72	Wogonoside	C_22_H_20_O_11_	M+H	461.10784	461.10800	0.3	461[M+H]^+^,285[M+H-C_6_H_8_O_6_]^+^,270[M+H-C_6_H_8_O_6_-CH_3_]^+^
18	3.76	Oxystemoninine	C_22_H_33_NO_6_	M+H	408.23806	408.23762	−1.1	408[M+H]^+^,362[M+H−CH_2_O_2_]^+^,322[M+H−C_4_H_6_O_2_]^+^
19	3.80	Scutellarin	C_21_H_18_O_12_	M−H	461.07255	461.07244	−0.2	461[M−H]^−^,285[M−H−C_6_H_8_O_6_]^−^
20	3.80	Tuberostemoninol A	C_22_H_31_NO_6_	M+H	406.22241	406.22281	1.0	406[M+H]^+^,388[M+H−H_2_O]^+^,360[M+H−H_2_O−CO]^+^,200[M+H−C_11_H_12_NO_3_]^+^,182[M+H−C_11_H_12_NO_3_−H_2_O]^+^
21	3.94	Tuberostemonine	C_22_H_33_NO_4_	M+H	376.24824	376.24807	−0.5	376[M+H]^+^,302[M+H−C_3_H_6_O_2_]^+^
22	4.00	Stemoninine A/B	C_22_H_29_NO_5_	M+H	388.21185	388.21174	−0.3	388[M+H]^+^,221[M+H−C_9_H_13_NO_2_]^+^,182[M+H−C_9_H_13_NO_2_−C_3_H_3_]^+^
23	4.15	Baicalcin	C_15_H_10_O_5_	M−H	269.04555	269.04506	−1.8	269[M−H]^−^,213[M−H−H_2_O−C_2_HO]^−^,197[M+H−2H_2_O−C_2_HO]^−^
24	4.76	Skullcapflavone Ⅱ	C_19_H_18_O_8_	M+H	375.10744	375.10749	0.1	375[M+H]^+^,345[M+H−2CH_3_]^+^,327[M+H−2CH_3_−H_2_O]^+^
25	4.78	Skullcapflavone I	C_17_H_14_O_6_	M+H	315.08631	315.08661	0.9	315[M+H]^+^,300[M+H−CH_3_]^+^,285[M+H-CH_2_O]^+^,257[M+H−CH_2_O−CO]^+^,182[M+H−CH_2_O−C_7_H_3_O]^+^,154[M+H−C_8_H_5_O_2_−CO]^+^
26	4.81	Tectorigenin	C_16_H_12_O_6_	M+H	301.07066	301.07038	−1.0	301[M+H]^+^,286[M+H−CH_3_]^+^,183[M+H−C_8_H_6_O]^+^
27	5.09	Aurantio-obtusin	C_17_H_14_O_7_	M+H	331.08123	331.07986	−4.1	331[M+H]^+^,301[M+H−2CH_3_]^+^
28	5.24	luteolin	C_15_H_10_O_6_	M−H	285.04046	285.04019	−1.0	285[M−H]^−^,151[M−H−C_8_H_6_O_2_]^−^,133[M−H−C_8_H_6_O_2_−H_2_O]^−^,125[M−H−C_9_H_4_O_3_]^−^
29	5.63	Riboderm	C_17_H_20_N_4_O_6_	M+Na	399.12751	399.12951	5.0	399[M+Na]^+^,319[M−3H_2_O−CO]^+^,212[M+H−C_7_H_9_NO_5_]^+^,184[M+H−C_7_H_9_NO_5_−CO]^+^
30	6.12	Guanosine	C_10_H_13_N_5_O_5_	M+Na	306.08089	306.08132	1.4	306[M+Na]^+^,246[M−C_2_H_4_O_2_]^+^,204[M−C_2_H_4_O_2_−C2H_2_O]^+^
31	7.23	Didehydrotuberostemoninee	C_22_H_29_NO_4_	M+H	372.21694	372.21669	−0.7	372[M+H]^+^,326[M+H−CH_2_O_2_]^+^,308[M+H−CH_2_O_2_−H_2_O]^+^

### Inference of Typical Compound Cleavage Law

The components that enter the lungs and blood are mostly flavonoids and alkaloids. Flavonoids and alkaloids have strong effects of inhibiting microorganisms, antioxidation, anti-inflammatory, protecting blood vessels, and neuroprotection (Birt et al., 2001; Parnell et al., 2012; Meng et al., 2014; Xie F. et al., 2020; Ku et al., 2020; Rui et al., 2020; Porras et al., 2021). Take the flavonoid “wogonin” and the alkaloid “tuberostemoninol A” as examples to illustrate the identification process.

**FIGURE 6:**
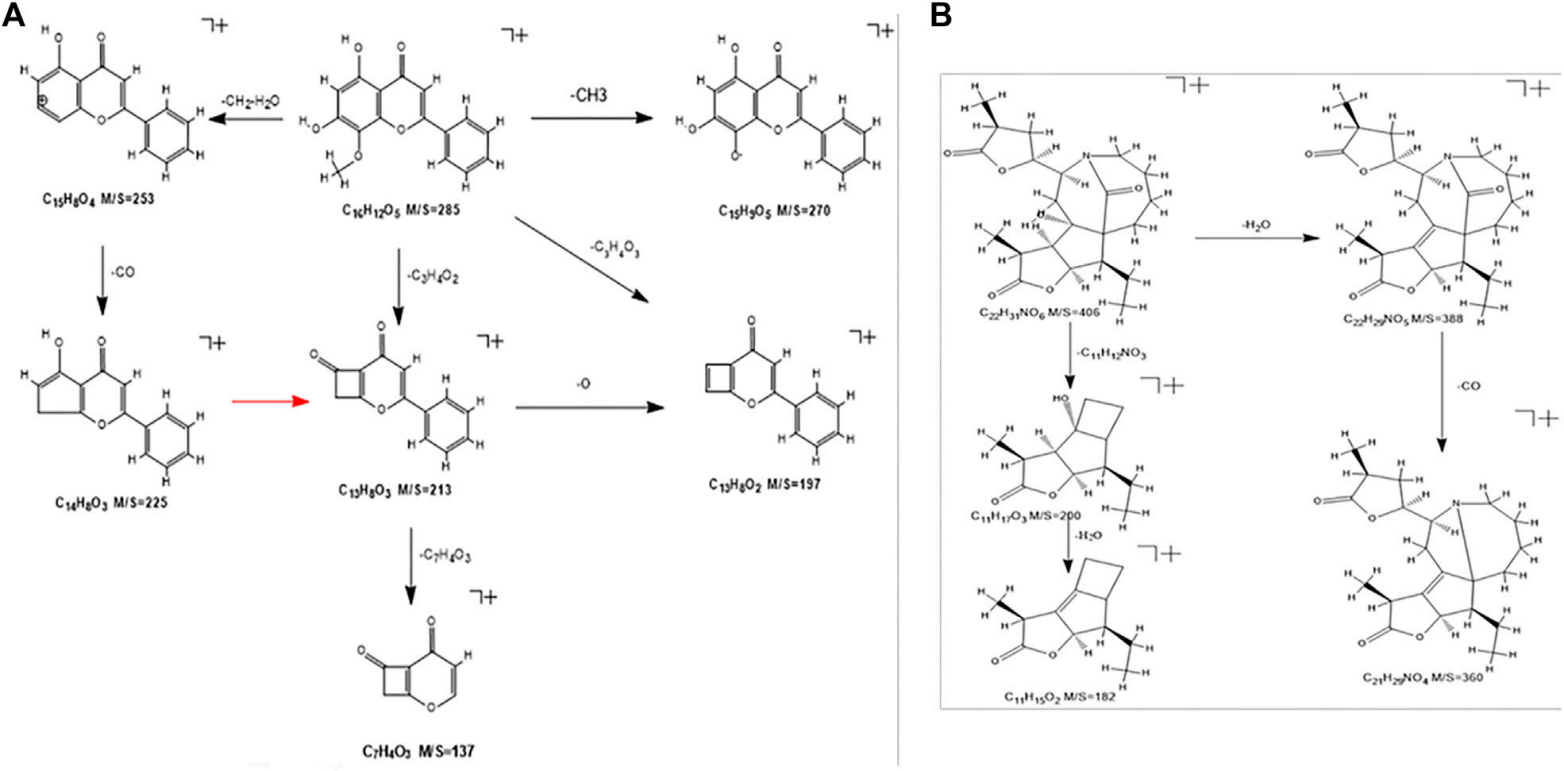
**(A)** Wogonin’s cracking method. **(B)** The cracking method of Tubeostemoninol A.

The wogonin quasi-molecular ion is m/z 285[M+H]^+^. This fragment has a relatively high abundance, and the fragmentation is divided into three ways. The quasi-molecular ion A-ring removes the methyl group attached to the methoxy group, and the oxygen forms at one end of the ion to form an end that connects to the benzene ring. Another cleavage pathway is the combination of the hydroxyl group of the A-ring and the methyl hydrogen to remove a molecule of water, and the remaining ethyl group will then fall off to form the fragment ion of m/z253. Continue to remove the carbonyl group to form m/z225 fragment ions. The third way is that under the bombardment of high-energy particles, the A-ring is connected with the methoxy group and the hydroxyl group to break off the carbon atoms to form a ring-opening rearrangement to form m/z213 fragment ions and continue to deoxidize to form m/z197 fragment ions or directly remove the B-ring benzene ring to form m/z137 fragments. The specific cracking method is shown in Figure 6A.

The cleavage of tuberostemoninol A is relatively simple. The two ways are first to continuously remove H_2_O and CO to form m/z388 and m/z360 fragment ions. The other is to remove two carbon rings to form m/z200 rearranged ions, and continue to remove water molecules to form m/z182 fragment ions. The specific cracking method is shown in Figure 6B.

### Target Analysis

414 (Four hundred and fourteen) component targets were obtained by predicting the component targets of the four databases. And one related gene chip was retrieved from the database (Chen JW. et al., 2020): GSE40012 is provided by the Intensive Care Lab of the Nepean Clinical School of University of Sydney, Kingswood, NSW, Australia. The chip contains 190 gene expression data sets of influenza pneumonia patients, bacterial pneumonia patients, mixed bacterial and influenza pneumonia patients, systemic inflammatory response patients, and healthy control. We selected 61 cases of bacterial pneumonia patients and 36 cases of healthy control, a total of 97 cases for research. Use impute package v1.62.0 and limma package v3.44.3 of R v4.0.0 to impute missing values, standardize, and determine differential genes through empirical Bayesian models. The threshold of differential genes is set to *p* < 0.05 and |log2 fold change| ≥1. To ensure the accuracy of disease target prediction, the disease targets from the above sources were obtained by intersecting the disease targets, and 778 targets were obtained. Finally, the component targets and disease targets were re-intersected. Finally, 67 therapeutic targets were obtained (the above retrieval date ended at 2020.12.1).

### C-T Network and T-D Network and Topology Analysis

**FIGURE 7:**
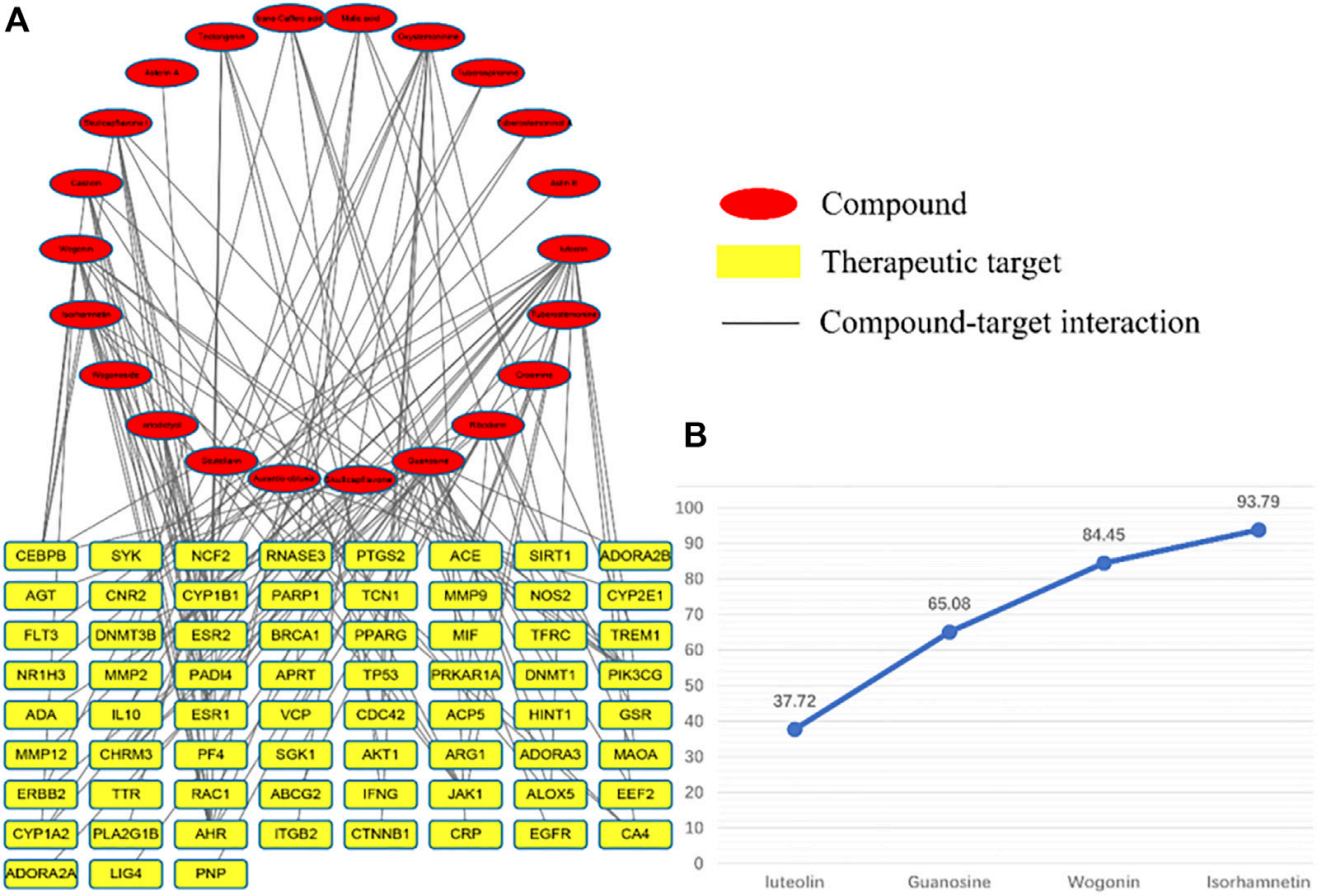
C-T network diagram, red represents components and yellow represents targets. **(A)** is the contribution of each component to the C-T network and **(B)** is the four core components that contribute more than 85%.

After the C-T network deletes the isolated nodes and their connected edges, there is a total of 89 nodes, representing 22 compound components and 67 therapeutic targets; 129 edges represent 129 component–target relationships (Figure 7). The results show that the total CI of luteolin, guanosine, wogonin, and isorhamnetin is 93.79% (greater than 85%), which is the core component that contributes the most to the C-T network.

**FIGURE 8:**
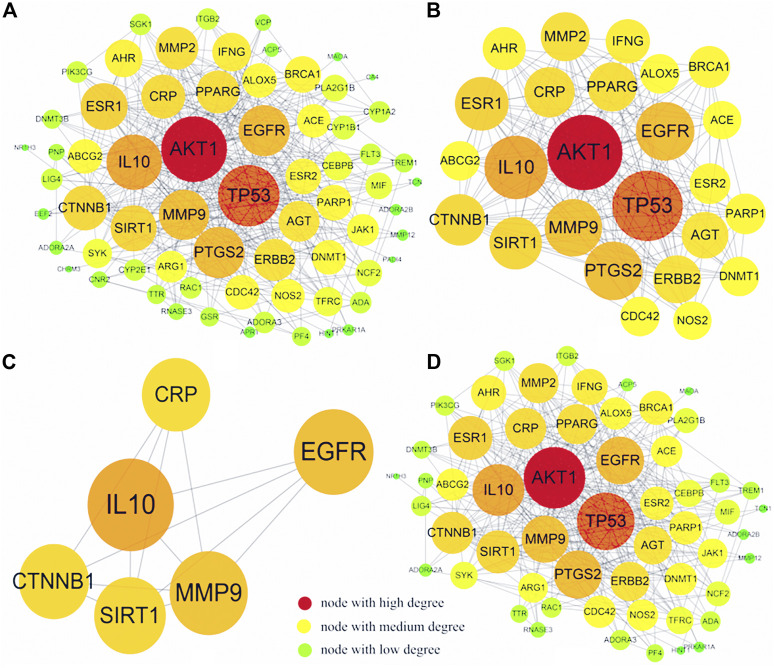
T-D network diagram. **(A)** is the graph after the isolated nodes and connected edges are deducted, **(B)** is the relevant information graph after extracting the mean value greater than a degree (11.463), **(C)** is the graph of six core targets, **(D)** is the six core targets The core target network of the remaining connecting nodes.

The T-D network finally screened and obtained six core therapeutic targets: SIRT1, IL-10, MMP9, CTNNB1, CRP, and EGFR (Table 3). The therapeutic target and the interaction relationship between the targets are imported into Cytoscape to construct a T-D network. After deleting the isolated nodes and connected edges, there are 67 nodes and 384 edges in the network (Figure 8A). Extract the target points greater than the mean value of the degree (11.463) to form the core target point network 1. After deleting the isolated nodes and connected edges, there are 25 nodes and 184 edges in the network (Figure 8B). Extract six core targets to form a core target network2. After deleting the isolated nodes and connected edges, there are six nodes and 13 edges in the network (Figure 8C). Extract six core targets and their directly connected nodes to form a core target network 3, which has 56 nodes and 341 edges (Figure 8D). There are only six core targets, but they are connected to 83.58% of the nodes (56/67) and 88.80% of the edges (341/384) of the T-D network. It shows that these six nodes play an extremely important role in maintaining the stability of the T-D network.

**TABLE 3:** Core targets and correlation coefficients.

name	AverageShortestPathLength	Betweenness	Closeness	Degree	Coreness
SIRT1	1.65151515	0.09071521	0.60550459	24	9
IL-10	1.53030303	0.09502362	0.65346535	33	9
MMP9	1.60606061	0.07128803	0.62264151	28	9
CTNNB1	1.68181818	0.03984413	0.59459459	23	9
CRP	1.68181818	0.04837266	0.59459459	22	8
EGFR	1.57575758	0.04708932	0.63461538	29	8

### Molecular Docking Verification

**FIGURE 9:**
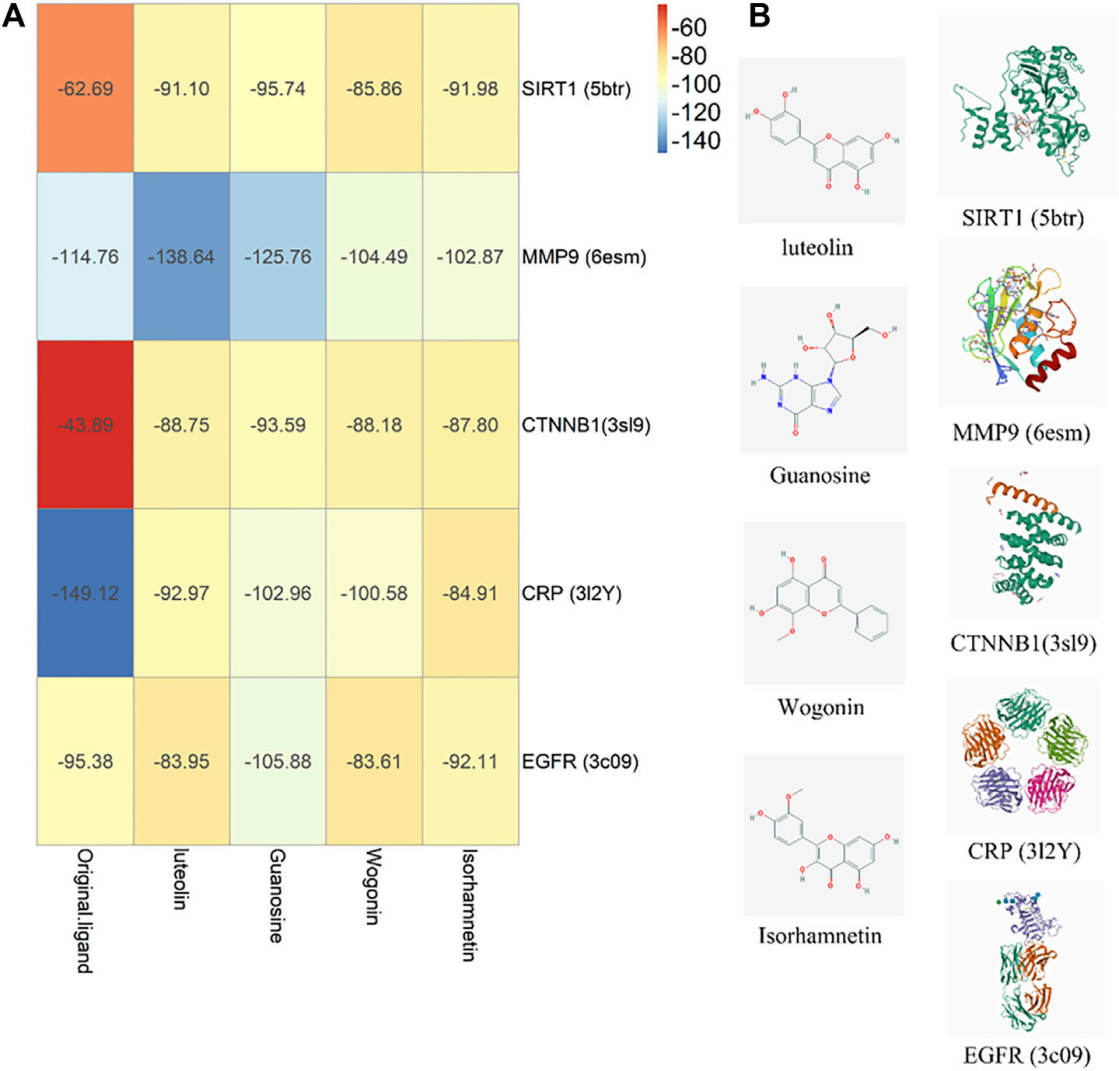
Molecule docking verification diagram. **(A)** Component-target affinity energy heat map, the greater the absolute value of kJ/mol, the stronger the binding energy. **(B)** Molecule docking diagram.

The four core components luteolin, guanosine, wogonin, and isorhamnetin and the six core targets SIRT1, IL-10, MMP9, CTNNB1, CRP, and EGFR were screened for molecular docking according to their binding affinity. It is generally believed that the absolute value of the connection affinity energy value is close to or greater than the original protein docking energy value, which means that the docking is completed. In this experiment, good results were obtained except for CRP and the protein configuration of IL-10 was not found in the database, considering that it plays a significant role as a signal factor involved in inhibiting inflammation, promoting humoral immunity, and tissue repair (Hendriks et al., 2004; Zhang et al., 2019; Singh et al., 2020). Finally, as shown in Figure 8, it demonstrated good binding ability (Figure 9A). The docking effect between the core target and the core component is shown in Figure 9B.

### Gene and Biomarker Enrichment Analysis

**FIGURE 10:**
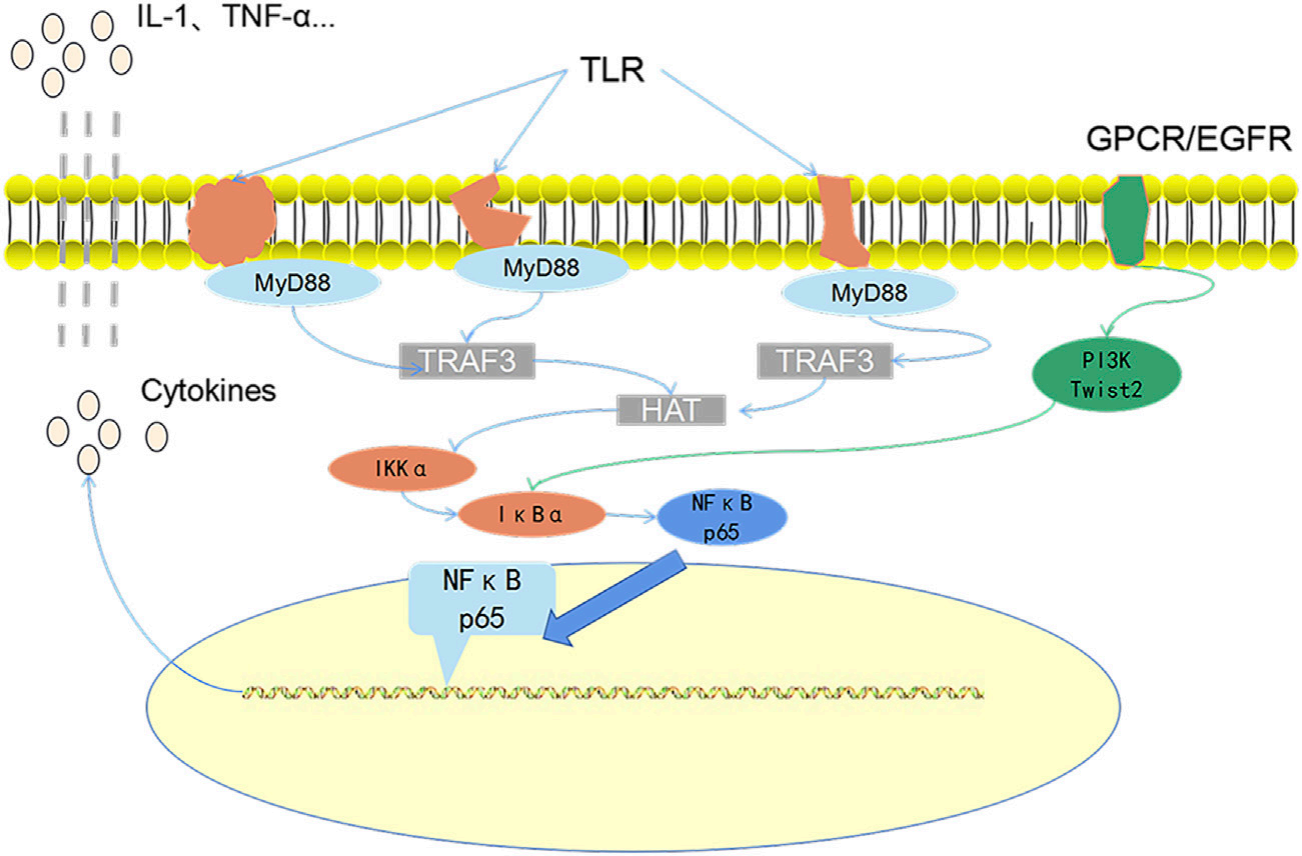
The core role path diagram. MP is specifically recognized by toll-like receptors and then promotes the activation of the NF-κB pathway by promoting the expression of MyD88 protein. This is also the main path of MPP pathogenesis. Both G-protein–coupled receptors and EGF receptors that can pass the direct or indirect pathway is positively correlated with the NF-κB pathway.

The selected core biomarkers and core target enrichment analysis are integrated with pathway over-representation analysis. The biologically significant pathways satisfying *p* < 0.05 and Q < 0.05 are shown in the following table (Table 4). Through the combined analysis of metabolomics and network pharmacology, it can be seen that QB mainly interfere with the G alpha (q) signaling events, GPCR downstream signaling, signal transduction, signaling by GPCR, spinal cord injury, GnRH signaling pathway, relationship between inflammation COX-2 and EGFR, and other interventions in the disease process, mainly the intervention of G-protein–coupled receptors, and EGFR. Through the analysis of the combined path of the two, G-protein–coupled receptors can directly promote the activation of the PI3K pathway (Li et al., 2021), while EGFR directly promotes the expression of Twist2 (Lehman et al., 2020). Both the STAT3 and PI3K pathways act as the direct upstream of the NF-κB pathway, directly or through Twist2, promoting the activation of the NF-κB pathway (Figure 10).

**TABLE 4:** Enrichment analysis chart.

Pathway	Overlapping genes	Num all pathway genes	Overlapping metabolites	Num all pathway metabolites	P Joint	Q joint
G-alpha (q) signaling events	EGFR	221 (221)	HMDB00181; HMDB00220; HMDB00674; HMDB01388; HMDB00806; HMDB60102; HMDB01043; HMDB00207; HMDB01999; HMDB02183	56 (64)	2.99E−07	7.44E−05
GPCR downstream signaling	EGFR	633 (633)	HMDB00181; HMDB00220; HMDB00674; HMDB00564; HMDB01388; HMDB00806; HMDB60102; HMDB01043; HMDB00207; HMDB01999; HMDB02183	118 (152)	4.78E−05	0.00327
Signal transduction	CTNNB1; EGFR; MMP9	2431 (2432)	HMDB00181; HMDB00220; HMDB00674; HMDB00222; HMDB00564; HMDB01388; HMDB00806; HMDB60102; HMDB01043; HMDB00207; HMDB01999; HMDB02183	206 (290)	0.000224	0.0119
Signaling by GPCR	EGFR	706 (706)	HMDB00181; HMDB00220; HMDB00674; HMDB00564; HMDB01388; HMDB00806; HMDB60102; HMDB01043; HMDB00207; HMDB01999; HMDB02183	142 (190)	0.000233	0.0119
Spinal cord injury	EGFR; MMP9	117 (117)	HMDB60102; HMDB01043	5 (9)	0.000646	0.0255
GnRH signaling pathway - Homo sapiens (human)	EGFR	93 (93)	HMDB00674; HMDB60102; HMDB01043	6 (6)	0.00135	0.0419
Relationship between inflammation_ COX-2 and EGFR	EGFR	25 (25)	HMDB60102; HMDB01043	1 (2)	0.00153	0.0442

### Western Blotting Detection

Through the analysis of the combined path of biomarkers and core disease targets, it is further obtained that its main function and the intracellular NF-κB pathway are found through further analysis, and TLR-MyD88-NF-κB p65 is the most critical signaling pathway for the pathogenesis and treatment of MPP. The TLR4/MyD88/NF-κB signaling pathway is involved in the body’s immune response and alveolar inflammation. TLR4 is activated after body injury, which further promotes the expression of downstream factors MyD88 and NF-κB and promotes the expression of inflammatory factors. This causes the alveolar epithelial cells to synthesize and secrete pulmonary inflammation–related cytokines and alveolar epithelial cell apoptosis, leading to the occurrence of pneumonia (Wang et al., 2020).

**FIGURE 11:**
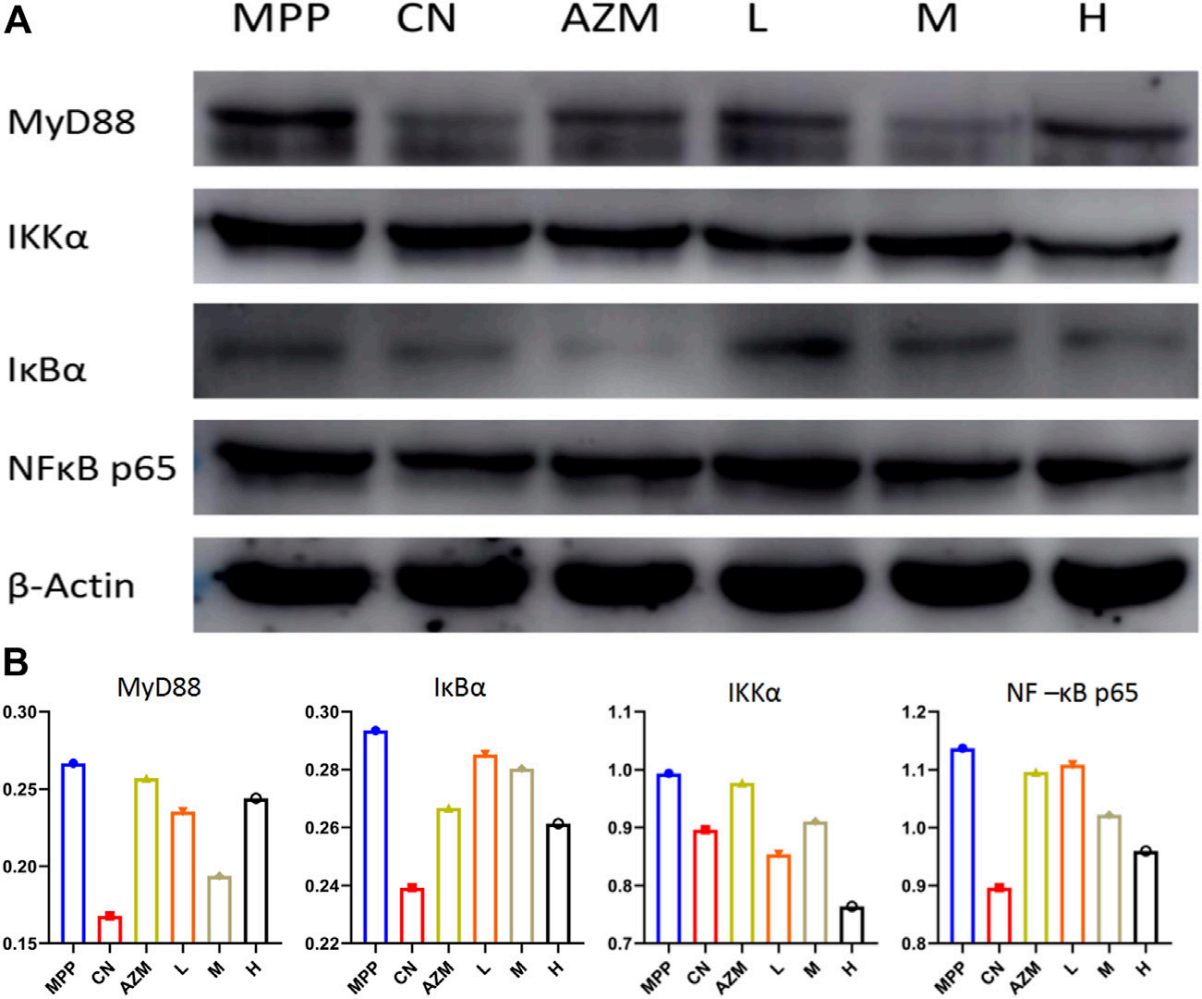
**(A)** Western blot map of each histone. **(B)** Gray value of each histone.

The protein expression detection was carried out according to the Western Blot method. The results are shown in Figure 11A. Compared with the blank group, the protein band of the model group was deepened and there was a trend of callback after administration. The callback trend of M and H is similar or better than that of the CN.

Using the gray value to analyze the protein expression level, the high dose has a better callback effect on NF-κB p65 than azithromycin (*p* < 0.05). The regulation of the upstream protein shows an obvious trend of superiority with increasing dose. The middle dose inhibited MyD88 the strongest. This reflects that a high dose has the advantage of strongly inhibiting the NF-κB pathway, but does not directly inhibit the agonistic ability of toll-like receptors. It may even have a certain activation ability. In this case, the middle dose is considered the optimal dose (Figure 11B).

## Conclusion

Firstly, we verified that QB has a good ability to treat mycoplasma pneumonia through pharmacodynamic evaluation. Then, we identified the main biomarkers of its action. Through the study of the rise and fall trends of these biomarkers, it can be proved that MPP causes the abnormal changes of the original biological small molecules in internal medicine. QB can regulate some substances, proving its ability to treat MPP. Subsequently, through extreme drug delivery, the compounds that can be directly absorbed and utilized are characterized, and the main action components and main disease treatment targets are identified through network pharmacology technology. The final disease treatment is determined through the verification of the molecular docking between the components and the target. The drug first acts on the target and regulates the body through the signal transduction pathway in the body. The final reflection is the changes in proteins and endogenous small molecules. In MPP, it is the recovery of inflammatory factor levels and biomarker levels. Finally, through the enrichment of quality markers and targets, the pathway was further comprehensively analyzed. It is also confirmed that MyD88/NF-κB is the main signal pathway for QB intervention and treatment of MPP. The verification and confirmation of the protein expression of QB-related pathways in the lung tissue can significantly inhibit the activation of this pathway, thereby regulating downstream signal factors.

Both QB and AZM inhibited the survival of *Mycoplasma* in the lungs, restored normal immune levels, and repaired the damaged lungs. Compared with AZM, QB not only has a good ability to inhibit mycoplasma in the body but also has a better repair ability for inflammation infiltration. For long-lasting diseases caused by inflammatory damage, QB can inhibit pathogens while exerting a certain ability to promote body healing. Using the tracheal intubation technology to create the MPP model, the rat immune system has unusually strong resistance. The objectively evaluated physiological and biochemical indicators have evidence that both AZM and QB have shown strong strength in fighting pathogens. One question that needs to be considered is whether it is necessary to directly kill the pathogen while facing the invasion of some pathogens and whether some auxiliary means are needed to prevent the worsening of the disease. The prognosis is even more important. Preventing the persistent and repetitive development of inflammation is the key. We need to provide safe and reliable drugs to protect life and health, which requires us to evaluate drugs objectively and accurately. The application of safe and effective natural medicine adjuvant treatment is also key means to avoid antibiotic resistance.

The invasion of MP activates the body’s innate immune system and the toll-like receptor 2 (TLR2) on immune cells that specifically bind to *Mycoplasma pneumoniae*. Then, inflammatory infiltration occurs, which stimulates the secretion of pro-inflammatory cytokines, interleukins, and chemokines (Cronin et al., 2012). Since the mucosa is the first barrier to pathogen infection, research on the signal cascade and subsequent expression of inflammatory mediators is the key to studying the occurrence and development of diseases and drug treatment. The detection of pathogens in mammals depends on the recognition of pathogen-associated molecular patterns (PAMP) through pattern recognition receptors on cells of the innate immune system. The main family of pattern recognition receptors is ten toll-like receptors (TLR1-10) (Takeuchi and Akira, 2010). TLR2 and TLR4 specifically recognize MP and then through MyD88-dependent pathways. This leads to the recruitment, activation, and degradation of IRAK-1, degradation of the downstream protein IκB kinase (IKK) and NF-κB activation, which stimulates the NF-κB activation expression of pro-inflammatory cytokines (IL-1β, IL-6, IL-8, and TNF-α) (Lye et al., 2004; Shimizu, 2016; Chen J. et al., 2020). NF-κB is a ubiquitous transcription factor with a wide range of target genes, mainly intervening in inflammation and immune processes. MPP causes the first activation of MyD88, and the recruitment of MyD88 is a prerequisite for inflammatory signal transduction and the convergence of a variety of pro-inflammatory cytokines. It is the unique target of severe pro-inflammatory cytokine signaling intervention (Saikh, 2021). The high expression of MyD88 also confirms the activation of TLRs (except TLR3). The inhibition of MyD88 homodimerization is a prerequisite for the inhibition of its downstream inflammatory pathway expression. After sending a signal to cause phosphorylation of IKκ, IκB is degraded by the proteasome and NF-κB subunits p65 and p50 translocate to the nucleus (Chuffa et al., 2015). Therefore, it can be considered that MyD88 has the same trend with the entire NF-κB pathway. MPP caused an increase in the expression of MyD88 protein, reflecting the activation of the entire toll-like receptor. We noticed that AZM has a weak direct inhibitory ability on MyD88 while QBM (0.16 g/ml) has a better effect on the entire signal pathway. Inhibition ability and downstream inflammatory factors also reflect this fact.

The biomarkers of metabolomics are mainly concentrated in lipid metabolism and amino acid metabolism. By promoting the expression of NF-κB, it accelerates the response to pro-inflammatory factors, including TNF-α release (Ding et al., 2021), while reducing the secretion of NO, iNOs, etc., (Lowry et al., 2020), reflecting the duality of lipid metabolism. The pro-inflammatory effect of lipid metabolism is reflected off the first invasion of pathogens. Molecules including arachidonic acid and linoleic acid combine with lipoxygenase to promote the secretion of IL-6 and TNF-α by macrophages through the production of prostaglandins and leukotrienes (Das, 2010; Poorani et al., 2016). When the inflammation invasion reaches the second stage, it can promote the conversion to macrophages from the M1 type to M2 type and promote the release of IL-4, IL-10, etc (Das, 2018). In this experiment, whether it is the level of lipid metabolism or the Th1 and Th2 type, cytokines are highly expressed in the MPP group, which reflects metabolic disorders. The abnormality of amino acid metabolism is believed to be directly related to immune regulation. By regulating the phosphorylation of MyD88, inflammation is regulated to promote the release of TNF-α and IL-1β (Gurung et al., 2017).

SIRT1 can inhibit SREBP1 and further downregulate pirin (PIR) and nod-like receptor protein 3 (NLRP3) inflammasomes, deacetylating FOXO3, NF-κB, and p532 to achieve the purpose of inhibiting inflammation (Sun et al., 2009). It can promote synthesis inhibiting activity of eNOS oxidase slow oxidative stress (Zhang et al., 2020) and to protect lung tissue. MMP9 (matrix metallopeptidase 9) is a protease related to the reconstruction process of lung pathology. It interferes with the transport of eosinophils and is usually produced by a variety of airway cells, especially airway epithelial cells (Kong et al., 2014). MMP9 can be released from activated white blood cells, reduce the level of E-cadherin in lung tissue (Xie X. et al., 2020) and by intervening in the accumulation of neutrophils and eosinophils. It can promote the killing of pathogens and inhibit excessive inflammation (Lu et al., 2008; Kong et al., 2014). CTNNB1 is the gene encoding β-catenin and is a key part of the Wnt signaling pathway. It is essential for cell proliferation, differentiation, and function. It plays an important role in stem cell renewal and organ regeneration under normal physiological conditions. CTNNB1 stimulates the phosphorylation of β-catenin and the transfer inside and outside the cell and in this process stimulates T-cell factors to maintain the stability of the immune system (Ridker and Lüscher, 2014). CTNNB1 abnormal expression is often closely related to the development of tumors, for its role in pneumonia is also thought to be immune homeostasis or not the citizenry. The epidermal growth factor receptor (EGFR) can promote the repair of respiratory epithelial cells and promote the expression of inflammatory factors (Harada et al., 2011). EGFR proteins, while reducing mucus generated by adjusting IRFs and exogenous IFN-γ against the invasion of pathogens, appear to strengthen the inflammatory infiltrate in the process (Kalinowski et al., 2018).

Finally, the enrichment analysis of them found out their relationship with the NF-κB pathway and finally confirmed the relationship between them. The final protein expression verification confirmed the above conjecture. MPP triggers the activation of toll-like receptors and a series of downstream proteins and inflammatory signal factors are therefore expressed. Immune damage and pathogen damage simultaneously damage the lung tissue. QB, through the regulation of related targets after administration, ultimately affects the NF-κB pathway, resulting in changes in downstream biomarkers.

Based on the present observations, QB can inhibit the proliferation of MP in lung tissues. It acts on SIRT1, IL-10, MMP9, CTNNB1, EGFR, and other targets through luteolin, guanosine, wogonin, isorhamnetin, and other components. Finally, by inhibiting the NF-κB signaling pathway, it regulates the release of inflammatory factors and inhibits the excessive Th2 response, which has the effects of regulating metabolism, curing diseases, and repairing lung tissue.

## Data Availability

The raw data supporting the conclusions of this article will be made available by the authors, without undue reservation.

## References

[B1] Alishlash A. S. Atkinson T. P. Schlappi C. Leal S. M. Waites K. B. Xiao L. (2019). Mycoplasma Pneumoniae Carriage with De Novo Macrolide-Resistance and Breakthrough Pneumonia. Pediatrics 144 (4), e20191642. 10.1542/peds.2019-1642 31488697

[B2] Almagor M. Kahane I. Yatziv S. (1984). Role of Superoxide Anion in Host Cell Injury Induced by Mycoplasma Pneumoniae Infection. A Study in normal and Trisomy 21 Cells. J. Clin. Invest. 73 (3), 842–847. 10.1172/JCI111279 6323529 PMC425088

[B3] Atkinson T. P. Balish M. F. Waites K. B. (2008). Epidemiology, Clinical Manifestations, Pathogenesis and Laboratory Detection of Mycoplasma Pneumoniae Infections. FEMS Microbiol. Rev. 32 (6), 956–973. 10.1111/j.1574-6976.2008.00129.x 18754792

[B4] Bajantri B. Venkatram S. Diaz-Fuentes G. (2018). Mycoplasma Pneumoniae: A Potentially Severe Infection. J. Clin. Med. Res. 10 (7), 535–544. 10.14740/jocmr3421w 29904437 PMC5997415

[B5] Birt D. F. Hendrich S. Wang W. (2001). Dietary Agents in Cancer Prevention: Flavonoids and Isoflavonoids. Pharmacol. Ther. 90, 157–177. 10.1016/s0163-7258(01)00137-1 11578656

[B6] Blyth C. C. Gerber J. S. (2018). Macrolides in Children with Community-Acquired Pneumonia: Panacea or Placebo. J. Pediatr. Infect Dis Soc 7 (1), 71–77. 10.1093/jpids/pix083 29096010

[B7] Chen J. Ullah H. Zheng Z. Gu X. Su C. Xiao L. (2020b). Soyasaponins Reduce Inflammation by Downregulating MyD88 Expression and Suppressing the Recruitments of TLR4 and MyD88 into Lipid Rafts. BMC Complement. Med. Ther. 20 (1), 167. 10.1186/s12906-020-2864-2 32493316 PMC7268359

[B8] Chen J. W. Rice T. A. Bannock J. M. Bielecka A. A. Strauss J. D. Catanzaro J. R. (2020a). Autoreactivity in Naïve Human Fetal B Cells Is Associated with Commensal Bacteria Recognition. Science 369 (6501), 320–325. 10.1126/science.aay9733 32675374

[B9] Chuffa L. G. Fioruci-Fontanelli B. A. Mendes L. O. Ferreira Seiva F. R. Martinez M. Fávaro W. J. (2015). Melatonin Attenuates the TLR4-Mediated Inflammatory Response Through MyD88- and TRIF-dependent Signaling Pathways in an In Vivo Model of Ovarian Cancer. BMC Cancer 15, 34. 10.1186/s12885-015-1032-4 25655081 PMC4322437

[B10] Collis M. C. (2006). Integrative Pharmacology and Drug Discovery-Iis the Tide Finally Turning. Nat. Rev. Drug Discov. 5 (5), 377–379. 10.1038/nrd2036 16628199

[B11] Cronin J. G. Turner M. L. Goetze L. Bryant C. E. Sheldon I. M. (2012). Toll-like Receptor 4 and MYD88-dependent Signaling Mechanisms of the Innate Immune System Are Essential for the Response to Lipopolysaccharide by Epithelial and Stromal Cells of the Bovine Endometrium. Biol. Reprod. 86 (2), 51. 10.1095/biolreprod.111.092718 22053092 PMC4396703

[B12] Das U. N. (2018). Arachidonic Acid and Other Unsaturated Fatty Acids and Some of Their Metabolites Function as Endogenous Antimicrobial Molecules: A Review. J. Adv. Res. 11, 57–66. 10.1016/j.jare.2018.01.001 30034876 PMC6052656

[B13] Das U. N. (2010). Current and Emerging Strategies for the Treatment and Management of Systemic Lupus Erythematosus Based on Molecular Signatures of Acute and Chronic Inflammation. J. Inflamm. Res. 3, 143–170. 10.2147/JIR.S9425 22096364 PMC3218729

[B14] Ding Y. Wang Y. Li C. Zhang Y. Hu S. Gao J. (2021). α-Linolenic Acid Attenuates Pseudo-allergic Reactions by Inhibiting Lyn Kinase Activity. Phytomedicine 80, 153391. 10.1016/j.phymed.2020.153391 33113502

[B15] Gurung P. Fan G. Lukens J. R. Vogel P. Tonks N. K. Kanneganti T. D. (2017). Tyrosine Kinase SYK Licenses MyD88 Adaptor Protein to Instigate IL-1α-Mediated Inflammatory Disease. Immunity 46 (4), 635–648. 10.1016/j.immuni.2017.03.014 28410990 PMC5501252

[B16] Hamilos D. L. (2015). Drivers of Chronic Rhinosinusitis: Inflammation Versus Infection. J. Allergy Clin. Immunol. 136 (6), 1454–1459. 10.1016/j.jaci.2015.10.011 26654194

[B17] Harada C. Kawaguchi T. Ogata-Suetsugu S. Yamada M. Hamada N. Maeyama T. (2011). EGFR Tyrosine Kinase Inhibition Worsens Acute Lung Injury in Mice with Repairing Airway Epithelium. Am. J. Respir. Crit. Care Med. 183 (6), 743–751. 10.1164/rccm.201002-0188OC 20935109

[B18] Hendriks J. J. Alblas J. van der Pol S. M. van Tol E. A. Dijkstra C. D. de Vries H. E. (2004). Flavonoids Influence Monocytic GTPase Activity and Are Protective in Experimental Allergic Encephalitis. J. Exp. Med. 200 (12), 1667–1672. 10.1084/jem.20040819 15611292 PMC2212002

[B19] Hsieh W. Y. Zhou Q. D. York A. G. Williams K. J. Scumpia P. O. Kronenberger E. B. (2020). Toll-Like Receptors Induce Signal-specific Reprogramming of the Macrophage Lipidome. Cell Metab 32 (1), 128–143.e5. 10.1016/j.cmet.2020.05.003 32516576 PMC7891175

[B20] Kalinowski A. Galen B. T. Ueki I. F. Sun Y. Mulenos A. Osafo-Addo A. (2018). Respiratory Syncytial Virus Activates Epidermal Growth Factor Receptor to Suppress Interferon Regulatory Factor 1-dependent Interferon-Lambda and Antiviral Defense in Airway Epithelium. Mucosal Immunol. 11 (3), 958–967. 10.1038/mi.2017.120 29411775 PMC6431552

[B21] Kannan T. R. Baseman J. B. (2006). ADP-ribosylating and Vacuolating Cytotoxin of Mycoplasma Pneumoniae Represents Unique Virulence Determinant Among Bacterial Pathogens. Proc. Natl. Acad. Sci. U S A. 103 (17), 6724–6729. 10.1073/pnas.0510644103 16617115 PMC1458948

[B22] Kannan T. R. Hardy R. D. Coalson J. J. Cavuoti D. C. Siegel J. D. Cagle M. (2012). Fatal Outcomes in Family Transmission of Mycoplasma Pneumoniae. Clin. Infect. Dis. 54 (2), 225–231. 10.1093/cid/cir769 22052890 PMC3245726

[B23] Karimi M. G. Arababadi M. K. (2014). TLR3 Plays Significant Roles Against Hepatitis B Virus. Mol. Biol. Rep. 41 (5), 3279–3286. 10.1007/s11033-014-3190-x 24477590

[B24] Khoury T. Sviri S. Rmeileh A. A. Nubani A. Abutbul A. Hoss S. (2016). Increased Rates of Intensive Care Unit Admission in Patients with Mycoplasma Pneumoniae: A Retrospective Study. Clin. Microbiol. Infect. 22 (8), 711–714. 10.1016/j.cmi.2016.05.028 27297319

[B25] Koh Y. Y. Park Y. Lee H. J. Kim C. K. (2001). Levels of Interleukin-2, Interferon-Gamma, and Interleukin-4 in Bronchoalveolar Lavage Fluid from Patients with Mycoplasma Pneumonia: Implication of Tendency Toward Increased Immunoglobulin E Production. Pediatrics 107 (3), E39. 10.1542/peds.107.3.e39 11230620

[B26] Kong M. Y. Clancy J. P. Peng N. Li Y. Szul T. J. Xu X. (2014). Pulmonary Matrix Metalloproteinase-9 Activity in Mechanically Ventilated Children with Respiratory Syncytial Virus. Eur. Respir. J. 43 (4), 1086–1096. 10.1183/09031936.00105613 24311764 PMC4059407

[B27] Ku Y. S. Ng M. S. Cheng S. S. Wing-Yi L. A. Lo A. W. Xiao Z. (2020). Understanding the Composition, Biosynthesis, Accumulation and Transport of Flavonoids in Crops for the Promotion of Crops as Healthy Sources of Flavonoids for Human Consumption. Nutrients 12 (6), 1717. 10.3390/nu12061717 PMC7352743 32521660

[B28] Kumar S. (2018). Mycoplasma Pneumoniae: A Significant but Underrated Pathogen in Paediatric Community-Acquired Lower Respiratory Tract Infections. Indian J. Med. Res. 147 (1), 23–31. 10.4103/ijmr.IJMR_1582_16 29749357 PMC5967212

[B29] Lehman H. L. Kidacki M. Stairs D. B. (2020). Twist2 Is NFkB-Responsive when P120-Catenin Is Inactivated and EGFR Is Overexpressed in Esophageal Keratinocytes. Sci. Rep. 10 (1), 18829. 10.1038/s41598-020-75866-0 33139779 PMC7608670

[B30] Li H. Lou R. Xu X. Xu C. Yu Y. Xu Y. (2021). The Variations in Human Orphan G Protein-Coupled Receptor QRFPR Affect PI3K-AKT-mTOR Signaling. J. Clin. Lab. Anal. 35 (7), e23822. 10.1002/jcla.23822 34018631 PMC8275006

[B31] Li J. (2020). Introduction to Traditional Chinese Medicine[Compilation and Dissemination of ]. Zhonghua Yi Shi Za Zhi 50 (1), 21–27. 10.3760/cma.j.issn.0255-7053.2020.01.004 32564533

[B32] Lin J. J. Lin K. L. Chiu C. H. Hsia S. H. Wang H. S. Chou I. J. (2014). Antineuronal Antibodies and Infectious Pathogens in Severe Acute Pediatric Encephalitis. J. Child. Neurol. 29 (1), 11–16. 10.1177/0883073812461944 23143714

[B33] Liu J. Wang R. Fang M. (2020). Clinical and Drug Resistance Characteristics of Providencia Stuartii Infections in 76 Patients. J. Int. Med. Res. 48 (10), 300060520962296. 10.1177/0300060520962296 33081537 PMC7588764

[B34] Liu Y. Ye X. Zhang H. Xu X. Li W. Zhu D. (2009). Antimicrobial Susceptibility of Mycoplasma Pneumoniae Isolates and Molecular Analysis of Macrolide-Resistant Strains from Shanghai, China. Antimicrob. Agents Chemother. 53 (5), 2160–2162. 10.1128/AAC.01684-08 19273684 PMC2681541

[B35] Lowry J. R. Marshall N. Wenzel T. J. Murray T. E. Klegeris A. (2020). The Dietary Fatty Acids α-linolenic Acid (ALA) and Linoleic Acid (LA) Selectively Inhibit Microglial Nitric Oxide Production. Mol. Cell Neurosci 109, 103569. 10.1016/j.mcn.2020.103569 33161065

[B36] Lu J. Marnell L. L. Marjon K. D. Mold C. Du Clos T. W. Sun P. D. (2008). Structural Recognition and Functional Activation of FcγR by Innate pentraxinsStructural Recognition and Functional Activation of FcgammaR by Innate Pentraxins. Nature 456 (7224), 989–992. 10.1038/nature07468 19011614 PMC2688732

[B37] Lye E. Mirtsos C. Suzuki N. Suzuki S. Yeh W. C. (2004). The Role of Interleukin 1 Receptor-Associated Kinase-4 (IRAK-4) Kinase Activity in IRAK-4-Mediated Signaling. J. Biol. Chem. 279 (39), 40653–40658. 10.1074/jbc.M402666200 15292196

[B38] Meng Y. Huo J. Lu W. Wang X. Zhang J. Wang W. (2012). Modulation of P1 and EGF Expression by Baicalin. Int. J. Mol. Sci. 14 (1), 146–157. 10.3390/ijms14010146 23344025 PMC3565255

[B39] Meng Y. Yang Y. Lu W. Wang Y. Qian F. Wang X. (2014). The Inhibition of Platycodin D on Mycoplasma Pneumoniae Proliferation and its Effect on Promoting Cell Growth After Anti-mycoplasma Pneumoniae Treatment. Front Cell Infect Microbiol 4, 192. 10.3389/fcimb.2014.00192 25629010 PMC4292783

[B40] Meng Y. L. Wang W. M. Lv D. D. An Q. X. Lu W. H. Wang X. (2017). The Effect of Platycodin D on the Expression of Cytoadherence Proteins P1 and P30 in Mycoplasma Pneumoniae Models. Environ. Toxicol. Pharmacol. 49, 188–193. 10.1016/j.etap.2017.01.001 28073091

[B41] Merrell D. S. Falkow S. (2004). Frontal and Stealth Attack Strategies in Microbial Pathogenesis. Nature 430 (6996), 250–256. 10.1038/nature02760 15241423

[B42] Naghib M. Hatam-Jahromi M. Niktab M. Ahmadi R. Kariminik A. (2018). Mycoplasma Pneumoniae and Toll-like Receptors: A Mutual Avenue. Allergol. Immunopathol (Madr) 46 (5), 508–513. 10.1016/j.aller.2017.09.021 29331619

[B43] Narita M. (2009). Pathogenesis of Neurologic Manifestations of Mycoplasma Pneumoniae Infection. Pediatr. Neurol. 41 (3), 159–166. 10.1016/j.pediatrneurol.2009.04.012 19664529

[B44] Onozuka D. Hashizume M. Hagihara A. (2009). Impact of Weather Factors on Mycoplasma Pneumoniae Pneumonia. Thorax 64 (6), 507–511. 10.1136/thx.2008.111237 19318345

[B45] Parnell G. P. McLean A. S. Booth D. R. Armstrong N. J. Nalos M. Huang S. J. (2012). A Distinct Influenza Infection Signature in the Blood Transcriptome of Patients with Severe Community-Acquired Pneumonia. Crit. Care 16 (4), R157. 10.1186/cc11477 22898401 PMC3580747

[B46] Poorani R. Bhatt A. N. Dwarakanath B. S. Das U. N. (2016). COX-2, Aspirin and Metabolism of Arachidonic, Eicosapentaenoic and Docosahexaenoic Acids and Their Physiological and Clinical Significance. Eur. J. Pharmacol. 785, 116–132. 10.1016/j.ejphar.2015.08.049 26335394

[B47] Porras G. Chassagne F. Lyles J. T. Marquez L. Dettweiler M. Salam A. M. (2021). Ethnobotany and the Role of Plant Natural Products in Antibiotic Drug Discovery. Chem. Rev. 121 (6), 3495–3560. 10.1021/acs.chemrev.0c00922 33164487 PMC8183567

[B48] Purba A. K. Ascobat P. Muchtar A. Wulandari L. Rosyid A. N. Purwono P. B. (2019). Multidrug-Resistant Infections Among Hospitalized Adults with Community-Acquired Pneumonia in an Indonesian Tertiary Referral Hospital. Infect. Drug Resist. 12, 3663–3675. 10.2147/IDR.S217842 31819549 PMC6883944

[B49] Qian W. Kang A. Peng L. Xie T. Ji J. Zhou W. (2018). Gas Chromatography-Mass Spectrometry Based Plasma Metabolomics of H1N1-Induced Inflammation in Mice and Intervention with Flos Lonicerae Japonica-Fructus Forsythiae Herb Pair. J. Chromatogr. B Analyt Technol. Biomed. Life Sci. 1092, 122–130. 10.1016/j.jchromb.2018.05.047 29890405

[B50] Ridker P. M. Lüscher T. F. (2014). Anti-inflammatory Therapies for Cardiovascular Disease. Eur. Heart J. 35 (27), 1782–1791. 10.1093/eurheartj/ehu203 24864079 PMC4155455

[B51] Rui Z. Chang-Pei X. Jing-Jing Z. Hong-Jun Y. (2020). Research Progress on Chemical Compositions of Coptidis Rhizoma and Pharmacological Effects of Berberine. Zhongguo Zhong Yao Za Zhi 45 (19), 4561–4573. 10.19540/j.cnki.cjcmm.20200527.202 33164419

[B52] Saikh K. U. (2021). MyD88 and beyond: A Perspective on MyD88-Targeted Therapeutic Approach for Modulation of Host Immunity. Immunol. Res. 69 (2), 117–128. 10.1007/s12026-021-09188-2 33834387 PMC8031343

[B53] Saraya T. (2017). Mycoplasma Pneumoniae Infection: Basics. J. Gen. Fam. Med. 18 (3), 118–125. 10.1002/jgf2.15 29264006 PMC5689399

[B54] Shimizu T. (2016). Inflammation-inducing Factors of Mycoplasma Pneumoniae. Front. Microbiol. 7, 414. 10.3389/fmicb.2016.00414 27065977 PMC4814563

[B55] Singh D. K. Dwivedi V. P. SinghPrakash. S. P. Kumari A. Sharma S. K. Ranganathan A. (2020). Luteolin-mediated Kv1.3 K+ Channel Inhibition Augments BCG Vaccine Efficacy Against Tuberculosis by Promoting Central Memory T Cell Responses in Mice. Plos Pathog. 16 (9), e1008887. 10.1371/journal.ppat.1008887 32956412 PMC7529197

[B56] Sun J. Madan R. Karp C. L. Braciale T. J. (2009). Effector T Cells Control Lung Inflammation during Acute Influenza Virus Infection by Producing IL-10. Nat. Med. 15 (3), 277–284. 10.1038/nm.1929 19234462 PMC2693210

[B57] Takeuchi O. Akira S. (2010). Pattern Recognition Receptors and Inflammation. Cell 140 (6), 805–820. 10.1016/j.cell.2010.01.022 20303872

[B58] Tang M. Xie X. Yi P. Kang J. Liao J. Li W. (2020). Integrating Network Pharmacology with Molecular Docking to Unravel the Active Compounds and Potential Mechanism of Simiao Pill Treating Rheumatoid Arthritis. Evid. Based Complement. Alternat Med. 2020, 5786053. 10.1155/2020/5786053 33204288 PMC7657688

[B59] Tibbitt C. A. Stark J. M. Mario. Martens L. Ma J. Mold J. E. Deswarte K. (2019). Single-Cell RNA Sequencing of the T Helper Cell Response to House Dust Mites Defines a Distinct Gene Expression Signature in Airway Th2 Cells. Immunity 51 (1), 169–184.e5. 10.1016/j.immuni.2019.05.014 31231035

[B60] Waites K. B. Balish M. F. Atkinson T. P. (2008). New Insights into the Pathogenesis and Detection of Mycoplasma Pneumoniae Infections. Future Microbiol. 3 (6), 635–648. 10.2217/17460913.3.6.635 19072181 PMC2633477

[B61] Waites K. B. Xiao L. Liu Y. Balish M. F. Atkinson T. P. (2017). Mycoplasma Pneumoniae from the Respiratory Tract and beyond. Clin. Microbiol. Rev. 30 (3), 747–809. 10.1128/CMR.00114-16 28539503 PMC5475226

[B62] Wang Y. Li H. Shi Y. Wang S. Xu Y. Li H. (2020). miR-143-3p Impacts on Pulmonary Inflammatory Factors and Cell Apoptosis in Mice with Mycoplasmal Pneumonia by Regulating TLR4/MyD88/NF-Κb Pathway. Biosci. Rep. 40 (7), BSR20193419. 10.1042/BSR20193419 32597476 PMC7340866

[B63] Wu H. Ding X. Zhao D. Liang Y. Ji W. (2019). Effect of Montelukast Combined with Methylprednisolone for the Treatment of Mycoplasma Pneumonia. J. Int. Med. Res. 47 (6), 2555–2561. 10.1177/0300060518820412 31072180 PMC6567689

[B64] Wynn T. A. (2015). Type 2 Cytokines: Mechanisms and Therapeutic Strategies. Nat. Rev. Immunol. 15 (5), 271–282. 10.1038/nri3831 25882242

[B65] Xie F. Zhang L. Yao Q. Shan L. Liu J. Dong N. (2020a). TUG1 Promoted Tumor Progression by Sponging miR-335-5p and Regulating CXCR4-Mediated Infiltration of Pro-tumor Immunocytes in CTNNB1-Mutated Hepatoblastoma. Onco Targets Ther. 13, 3105–3115. 10.2147/OTT.S234819 32341656 PMC7166065

[B66] Xie X. Shi Q. Wu P. Zhang X. Kambara H. Su J. (2020b). Single-cell Transcriptome Profiling Reveals Neutrophil Heterogeneity in Homeostasis and Infection. Nat. Immunol. 21 (9), 1119–1133. 10.1038/s41590-020-0736-z 32719519 PMC7442692

[B67] Xing Y. Wang D. Sheng K. Xiao X. Wei H. Liu L. (2020). Dynamic Change of Mycoplasma Pneumoniae Pneumonia in Hospitalized Children in a General Hospital: A 3-year Retrospective Analysis. Transl Pediatr. 9 (4), 522–531. 10.21037/tp-20-149 32953550 PMC7475305

[B68] Yamazaki T. Kenri T. (2016). Epidemiology of Mycoplasma Pneumoniae Infections in Japan and Therapeutic Strategies for Macrolide-Resistant M. Pneumoniae. Front. Microbiol. 7, 693. 10.3389/fmicb.2016.00693 27242718 PMC4876131

[B69] Yang H.-J. Song D. J. Shim J. Y. (2017). Mechanism of Resistance Acquisition and Treatment of Macrolide-Resistant Mycoplasma Pneumoniae Pneumonia in childrenMycoplasma pneumoniaeMechanism of Resistance Acquisition and Treatment of Macrolide-Resistant Pneumonia in Children. Korean J. Pediatr. 60 (6), 167–174. 10.3345/kjp.2017.60.6.167 28690643 PMC5500384

[B70] Yang M. Meng F. Gao M. Cheng G. Wang X. (2019). Cytokine Signatures Associate with Disease Severity in Children with Mycoplasma Pneumoniae Pneumonia. Sci. Rep. 9 (1), 17853. 10.1038/s41598-019-54313-9 31780733 PMC6882793

[B71] Zhang S. Weinberg S. DeBerge M. Gainullina A. Schipma M. Kinchen J. M. (2019). Efferocytosis Fuels Requirements of Fatty Acid Oxidation and the Electron Transport Chain to Polarize Macrophages for Tissue Repair. Cell Metab 29 (2), 443–456.e5. 10.1016/j.cmet.2018.12.004 30595481 PMC6471613

[B72] Zhang X. Zhang Y. Meng Q. Sun H. Wu S. Xu J. (2020). MicroRNA-382-5p Is Involved in Pulmonary Inflammation Induced by Fine Particulate Matter Exposure. Environ. Pollut. 262, 114278. 10.1016/j.envpol.2020.114278 32146367

[B73] Zhou Y. Zhang Y. Sheng Y. Zhang L. Shen Z. Chen Z. (2014). More Complications Occur in Macrolide-Resistant Than in Macrolide-Sensitive Mycoplasma Pneumoniae Pneumonia. Antimicrob. Agents Chemother. 58 (2), 1034–1038. 10.1128/AAC.01806-13 24277047 PMC3910883

